# Enrollee characteristics and receipt of colorectal cancer testing in Pennsylvania after adoption of the Affordable Care Act Medicaid expansion

**DOI:** 10.1002/cam4.6168

**Published:** 2023-06-17

**Authors:** Kirsten Y. Eom, Scott D. Rothenberger, Marian P. Jarlenski, Robert E. Schoen, Evan S. Cole, Lindsay M. Sabik

**Affiliations:** ^1^ Department of Medicine at the MetroHealth System at Case Western Reserve University Cleveland Ohio USA; ^2^ Division of General Internal Medicine, Department of Medicine University of Pittsburgh School of Medicine Pittsburgh Pennsylvania USA; ^3^ Department of Health Policy & Management University of Pittsburgh Graduate School of Public Health Pittsburgh Pennsylvania USA; ^4^ Division of Gastroenterology, Hepatology and Nutrition, Department of Medicine University of Pittsburgh School of Medicine Pittsburgh Pennsylvania USA

**Keywords:** colorectal cancer, medicaid administrative data, medicaid expansion, Pennsylvania, screening

## Abstract

**Background:**

Colorectal cancer (CRC) is the fourth most common cancer and the second leading cause of cancer‐related death in the U.S. Despite increased CRC screening rates, they remain low among low‐income non‐older adults, including Medicaid enrollees who are more likely to be diagnosed at advanced stages.

**Objectives:**

Given limited evidence regarding CRC screening service use among Medicaid enrollees, we examined multilevel factors associated with CRC testing among Medicaid enrollees in Pennsylvania after Medicaid expansion in 2015.

**Research Design:**

Using the 2014–2019 Medicaid administrative data, we performed multivariable logistic regression models to assess factors associated with CRC testing, adjusting for enrollment length and primary care services use.

**Subjects:**

We identified 15,439 adults aged 50–64 years newly enrolled through Medicaid expansion.

**Measures:**

Outcome measures include receiving any CRC testing and by modality.

**Results:**

About 32% of our study population received any CRC testing. Significant predictors for any CRC testing include being male, being Hispanic, having any chronic conditions, using primary care services ≤4 times annually, and having a higher county‐level median household income. Being 60–64 years at enrollment, using primary care services >4 times annually, and having higher county‐level unemployment rates were significantly associated with a decreased likelihood of receiving any CRC tests.

**Conclusions:**

CRC testing rates were low among adults newly enrolled in Medicaid under the Medicaid expansion in Pennsylvania relative to adults with high income. We observed different sets of significant factors associated with CRC testing by modality. Our findings underscore the urgency to tailor strategies by patients' racial, geographic, and clinical conditions for CRC screening.

## Graphical abstract

About 3 in 10 low‐income non‐elderly adults in Pennsylvania who newly gained Medicaid coverage under the ACA received any colorectal cancer (CRC) testing services. Being male, being Hispanic, having at least one chronic condition, using primary care services at least once a year, and having a higher county‐level median income are significant factors associated with an increased likelihood of receiving CRC testing services.

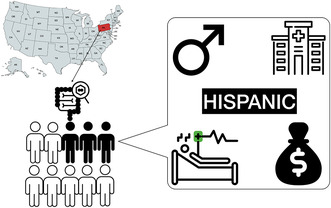

## 1 INTRODUCTION

Colorectal cancer (CRC) is the fourth most common cancer and the second leading cause of cancer death in the U.S.^1^ CRC incidence has been steadily decreasing over the past several decades largely due to improved uptake of guideline‐recommended CRC screening.^2^, ^3^, ^4^ Despite population‐wide improvements in CRC screening, disparities in CRC screening persist, especially among individuals with lower levels of income or education.^5^, ^6^, ^7^ In addition, race/ethnicity, age, sex, marital status, rurality of residence, health insurance coverage and type, having a usual source of care, and using primary care services are identified as significant factors associated with CRC screening.^5^, ^8^, ^9^, ^10^, ^11^ These factors are particularly relevant for adults enrolled in Medicaid programs, as many of them have low incomes and face socioeconomic barriers to accessing care. As a result, Medicaid enrollees are more likely to present with advanced‐stage cancers than individuals with private insurance or Medicare.^12^ Based on the 2012 Behavioral Risk Factor Surveillance System (BRFSS) and the 2013 National Health Interview Survey (NHIS) data, only 36% of Medicaid‐insured adults in the U.S. received guideline‐recommended CRC screening compared with at least 60% of privately or Medicare insured adults in 2013.^13^

In 2015, Pennsylvania implemented the Affordable Care Act (ACA), which provides funding for states to expand Medicaid eligibility to individuals <65 years with household incomes ≤138% of the federal poverty level (FPL).^14^ Pennsylvania also has a high burden of CRC as CRC is the fourth most common cancer and the second leading cause of cancer death,^7^ and annual CRC incidence and death rates were 9.1 and 7.6% higher than U.S. rates from 2000 to 2016, respectively.^15^ About 68% of age‐eligible Pennsylvanians reported lifetime any CRC screening in 2016, which increased by 2% from 2012.^16^ While factors associated with receipt of CRC screening among Medicare beneficiaries have been reported,^17^, ^18^, ^19^, ^20^ evidence regarding patterns of and factors associated with CRC screening among Medicaid enrollees is relatively limited.^11^, ^21^, ^22^, ^23^, ^24^, ^25^ As only about 45% of adults aged 50–64 years are up‐to‐date with CRC screening guidelines,^26^ identifying factors associated with CRC screening among this population is critical to reducing the overall burden of the disease. Furthermore, a rising CRC incidence rate among younger age groups in recent years underscores the need for a more comprehensive understanding of patterns of CRC screening among non‐elderly adults who are not yet age‐eligible for Medicare.^27^

This study aims to evaluate multilevel factors associated with CRC testing among Medicaid enrollees in the post‐ACA Medicaid expansion in Pennsylvania, one of the most populous states and largest Medicaid programs.

## 2 METHODS

### 2.1 Data sources

We used Pennsylvania Medicaid enrollment files, administrative claims, and encounter data from 2014 to 2019 provided by the Pennsylvania Department of Human Services. Enrollment files include age, gender, race/ethnicity, rurality of residence, and basis of Medicaid eligibility. Professional claims were used to create variables indicating CRC testing services and primary care services utilization based on Current Procedure Terminology (CPT) or Healthcare Common Procedural Coding System (HCPCS) procedure codes (Table S2). Inpatient, long‐term care, outpatient, professional, and pharmacy claims were used to compute the number of chronic conditions determined by the CMS Chronic Condition Warehouse (CCW) based on ICD‐9 and ICD‐10 codes (Table S3).^28^ We also use the Area Health Resources Files (AHRF) for county‐level socioeconomic and healthcare infrastructure data.^29^

### 2.2 Study population and study period

**FIGURE 1:**
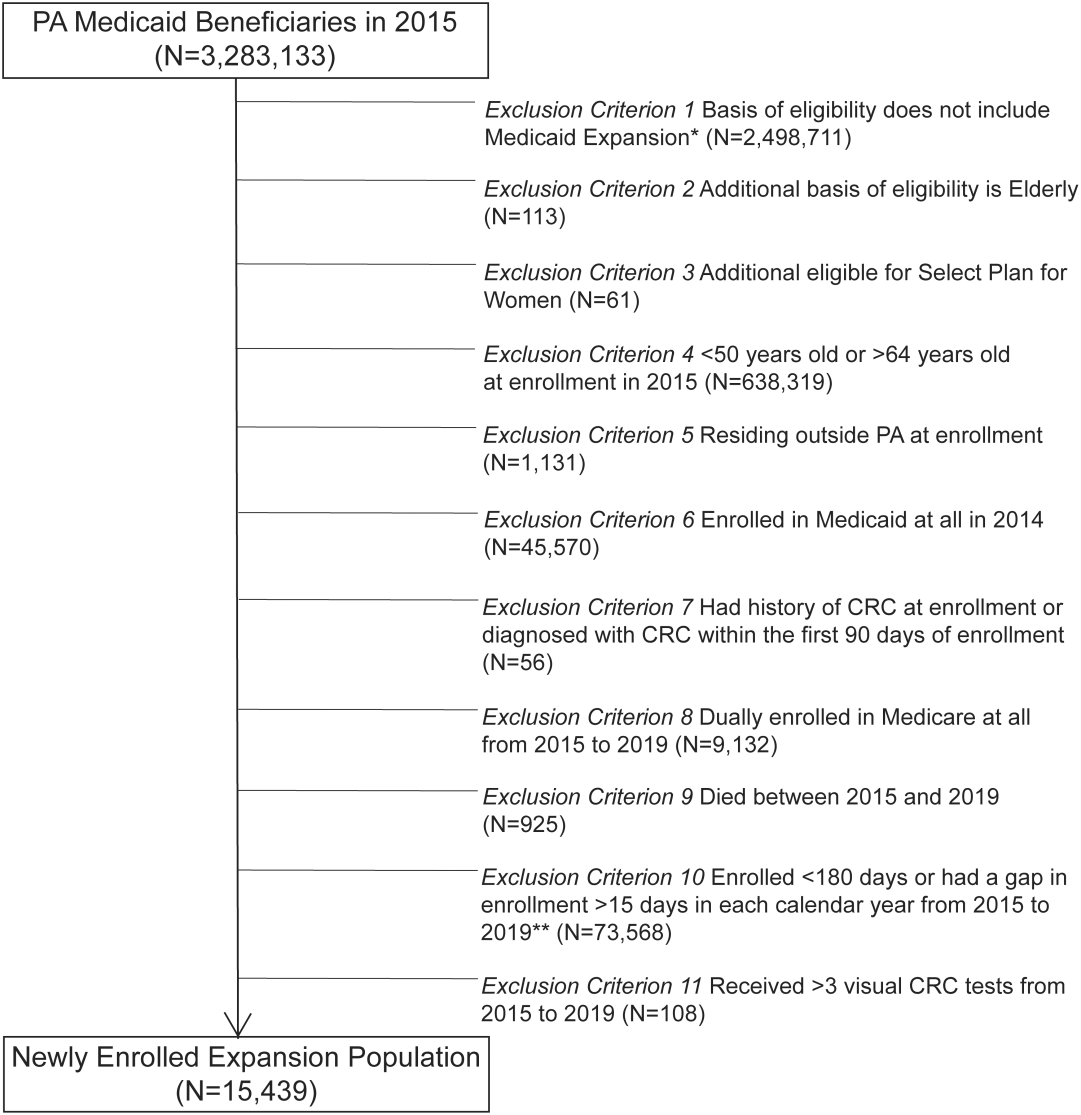
Study population inclusion and exclusion criteria. *We allowed multiple bases of eligibility if one of which was Medicaid expansion. **A gap in enrollment <15 days was considered as no gap. CRC, Colorectal Cancer.

The study population includes full‐benefit Medicaid enrollees aged 50–64 with a valid Pennsylvania ZIP code who were newly enrolled in Medicaid via Medicaid expansion in 2015 and continuously enrolled through 2019. We created a longitudinal cohort of newly enrolled adults aged 50–64 years in 2015 (the first year of Medicaid expansion in Pennsylvania) who were likely to have been uninsured prior to 2015 and identified the enrollees who were enrolled continuously, and thus, more likely to establish relationships with providers and receive care. We define continuous enrollment as enrollment for ≥180 days with no gap of <15 days in enrollment in each calendar year during the study period. In addition, we excluded enrollees with a history of CRC at enrollment or diagnosed with CRC within the first 90 days of enrollment.^30^ Figure 1 presents the derivation of the final study population of 15,439 enrollees. Any CRC tests include stool‐based tests (gFOBT, FIT, or FIT‐DNA) or visual tests (standard colonoscopy, flexible sigmoidoscopy, or CT colonography). Enrollees who received any CRC tests include 393 who received both stool‐based tests and visual tests during the study period.

### 2.3 Outcome measures

The primary outcome measure identifies whether enrollees had any CRC testing during the study period (Table S1), which is indicated as *Any CRC Tests*. The secondary outcome measure identifies receipt of CRC testing by modality: stool‐based test (guaiac fecal occult blood test (gFOBT) or fecal immunochemical test (FIT) or FIT‐DNA), which is indicated as *Stool‐based Test Alone*, or any visual testing (standard colonoscopy or flexible sigmoidoscopy or CT colonography), which is indicated as *Visual Tests Alone*.

### 2.4 Independent variables

Individual‐level sociodemographic independent variables include: age at enrollment, sex, race/ethnicity, rurality of residence based on the 2013 Rural–Urban Continuum Codes (RUCC),^31^ additional eligibility criteria at enrollment, state regions (results shown in Supplemental Document), total enrollment length per calendar year, the total number of chronic conditions at enrollment (Table S4),^30^ and frequency of primary care services utilization per calendar year. Regarding the rurality of residence, we defined metro counties of <1 million population as counties in metro areas of 250,000 to 1 million or counties in metro areas of fewer than 250,000. We defined non‐metro counties as counties with an urban population of 20,000 or more, adjacent to a metro area; urban population of 2500 to 19,999, adjacent to a metro area; urban population of 2500 to 19,990, not adjacent to a metro area; completely rural or <2500 urban population, adjacent to a metro area; or, completely rural or <2500 urban population, not adjacent to a metro area; and, urban areas with populations ranging from 2500 to 49,999 that are not part of larger labor market areas. We defined primary care services as services whose billing codes describe evaluation and management and wellness visits (Table S5) provided at physicians' offices, federally qualified health centers (FQHCs), rural health clinics (RHCs), outpatient hospitals, independent clinics, or public health clinics (Table S6). Regarding the frequency of primary care services, we translated multiple claims of primary care services on the same day into a single claim by prioritizing FQHCs/RHCs over other sites of care if they visited both FQHCs/RHCs and physician's office, outpatient hospitals, independent clinics, or public health clinics; we prioritized non‐physician's office for the enrollees with multiple claims of primary care services on the same day if they visited both a physician's office and outpatient hospitals, independent clinics, or public health clinics. We counted the number of claims for primary care services prior to the first claim for CRC testing for the enrollees who received any CRC testing as this study examines the association between the frequency of primary care services use and CRC testing. We computed the following county‐level independent sociodemographic and healthcare capacity variables: median household income; percent living in urban areas; percent unemployed among those ≥16 years; percent below poverty; percent <high school diploma among those ≥25 years; percent non‐Hispanic White; and numbers of primary care physicians, specialists, short‐term general hospital beds, FQHCs or RHCs per 1000 persons.

### 2.5 Statistical analysis

Due to the frequent exit and reentry of Medicaid beneficiaries as their eligibility changes,^32^, ^33^ we estimated a propensity score for enrollment length, adjusting for different observed characteristics among Medicaid enrollees, and calculated an inverse probability weight (IPCW).^34^ In addition, we estimated another propensity score for primary care services use, adjusting for an enrollee's observed characteristics related to primary care services use during the study period and calculated an inverse probability weight (IPTW) as primary care services use is associated with the enrollee's pre‐existing conditions, prior knowledge, and exposure to preventive services. We then created a composite inverse propensity weighting score (IPSW) by multiplying the IPCW and the IPTW and implemented the IPSW in all the proposed statistical analyses^35^, ^36^, ^37^; details of each adjustment are reported in supplemental materials (Tables S4–S7 and Figures S1–S19).

We compared individual‐ and county‐level characteristics of our study population by receipt of CRC testing, which is across CRC‐testing related outcomes, using chi‐square test for non‐continuous variables and *t*‐test for continuous variables. We also estimated multivariable logistic regression models to assess factors associated with each outcome measure (receipt of any CRC testing, stool‐based tests alone, and visual tests alone). We repeated these models adjusting for baseline individual‐level characteristics (Table 2 Column 1s) and adjusting for both baseline individual‐ and county‐level characteristics (Table 2 Column 2s). All these analyses were conducted as the weighted study population using the IPSW.

## 3 RESULTS

Table 1 describes the weighted baseline individual‐ and county‐level characteristics of 15,439 Medicaid enrollees aged 50–64 years newly enrolled in Medicaid and continuously enrolled from 2015 to 2019 to present any significantly unadjusted different baseline characteristics by the outcome. Table S9 presents the unweighted baseline characteristics of the study population. The weighted proportion of those who received any CRC testing during the study period was about 32% (*n* = 4835); about 75% (*n* = 3643) had a standard colonoscopy. About 42% of the study population was between 50 and 54 years old at enrollment, which did not significantly differ by receipt of any CRC tests. A significantly larger proportion of enrollees were Hispanic (10.5% vs. 6.9%, *p*‐value<0.001) or of other racial/ethnic groups among those who had any CRC tests than those with no CRC tests. Compared to those with no CRC tests, we observed more male enrollees among those who received any CRC tests (52.5% vs. 45.0%, *p*‐value<0.001). Enrollees who had any CRC tests enrolled in Medicaid significantly longer (*p*‐value<0.001) than those with no CRC tests, as the proportion enrolled for >360 days per calendar year was larger by 8% than that among enrollees with no CRC tests. We observed that a significantly larger proportion of enrollees with no CRC tests had missing information about chronic conditions than those with any CRC tests (24.0% vs. 8.3%, *p*‐value<0.001), and a significantly larger proportion of enrollees with any CRC tests had ≥1 chronic condition than those with no CRC tests (*p*‐value<0.001). Enrollees who had any CRC tests utilized primary care services more frequently than enrollees with no CRC tests (31.6% vs. 54.2%, *p*‐value<0.001). County‐level demographic and healthcare capacity characteristics were comparable between enrollees with any CRC tests and those with no CRC tests.

**TABLE 1:** Weighted individual‐ and county‐level characteristics of newly enrolled expansion population among Medicaid enrollees in Pennsylvania.

		(1)	(2)	(3)	(4)
	Overall	No CRC tests	Any CRC tests	Stool‐based tests alone	Visual tests alone
*N*	15,439	10,604	4835	800	3643
Weighted %	100.00	67.95	32.05	6.16	26.85
Individual‐level characteristics					
Age					
50–54	37.97	42.15	43.35	42.38	44.31
55–59	50.10	35.18	38.17	40.97	37.67
60–64	11.92	22.66	18.47	16.65	18.20
Sex			***	*	**
Female	55.02	55.02	47.54	46.85	48.70
Male	44.98	44.98	52.46	53.15	51.30
Race/Ethnicity			***	*	**
Non‐Hispanic White	65.29	66.81	61.34	60.44	61.44
Non‐Hispanic Black	17.29	15.89	14.59	13.83	14.94
Hispanic	6.38	6.94	10.54	8.10	11.14
Other	11.04	10.36	13.52	17.63	12.48
Rurality of residence				**	**
Metro counties >1 million population	51.61	52.67	48.85	65.89	45.99
Metro counties <1 million population	35.48	35.71	39.90	24.50	42.18
Nonmetro counties	12.91	11.62	11.25	9.61	11.83
Additional eligibility criteria					
Children and families	5.16	5.48	5.40	3.46	6.05
Disabled	5.04	5.92	6.45	7.27	6.70
Chronically Ill	0.42	0.48	0.21	0.00	0.26
Healthy horizons	5.77	6.48	7.17	9.37	7.08
Total enrollment days per calendar year			***		**
180–340	17.38	17.35	16.20	19.75	15.79
340.01–350	15.74	18.66	14.09	12.78	14.20
350.01–360	24.05	26.81	24.14	23.63	25.02
360.01–365	42.84	37.18	45.57	43.84	44.98
Total number of chronic conditions			***	***	***
0	39.08	40.22	28.84	29.38	27.44
1–2	20.54	17.96	28.54	26.88	29.26
3–4	11.83	9.66	19.02	18.68	19.75
5+	9.53	8.14	15.35	11.70	16.07
Missing	19.03	24.02	8.25	13.36	7.47
Frequency of primary care services use per calendar year			***	***	***
0	47.36	54.28	31.58	20.78	32.54
1–2	17.35	19.54	43.69	50.63	42.22
2–4	12.87	10.97	13.77	15.87	13.98
5+	22.42	15.20	10.96	12.72	11.27
County‐level characteristics					
Median household income					*
	54,190.59 (53,668.23, 54,712.95)	54,593.02 (53,895.13, 55,290.91)	53,739.91 (53,100.04, 54,379.77)	54,760.19 (53,124.59, 56,395.79)	53,463.13 (52,734.03, 54,192.23)
Percent living in Urban areas				***	
	79.11 (78.08, 80.14)	79.39 (78.26, 80.52)	79.49 (78.01, 80.96)	85.51 (83.24, 87.78)	78.49 (76.91, 80.06)
Percent Non‐Hispanic White				*	
	73.17 (72.32, 74.02)	73.78 (72.86, 74.70)	73.29 (71.96, 74.61)	68.53 (65.38, 71.68)	74.03 (72.54, 75.51)
Percent unemployed among population of 16+ years					
	5.47 (5.43, 5.51)	5.44 (5.39, 5.49)	5.42 (5.35, 5.49)	5.51 (5.37, 5.66)	5.42 (5.35, 5.49)
Percent in living under poverty					*
	14.69 (14.44, 14.94)	14.45 (14.15, 14.74)	14.66 (14.27, 15.05)	15.29 (14.30, 16.28)	14.62 (14.19, 15.05)
Percent <high school diploma among population of 25+ years					
	11.84 (11.67, 12.01)	11.67 (11.46, 11.89)	11.92 (11.67, 12.17)	11.71 (11.95, 12.37)	11.99 (11.70, 12.27)
# of PCPs per 1000 persons				*	
	0.78 (0.77, 0.79)	0.78 (0.77, 0.80)	0.79 (0.77, 0.81)	0.82 (0.79, 0.85)	0.78 (0.76, 0.81)
# of Specialists per 1000 persons				***	
	2.46 (2.40, 2.53)	2.43 (2.35, 2.51)	2.57 (2.39, 2.73)	2.85 (2.68, 3.02)	2.53 (2.33, 2.74)
# of Short‐term general hospital beds per 1000 persons				**	
	2.87 (2.82, 2.92)	2.82 (2.76, 2.89)	2.96 (2.82, 3.10)	3.02 (2.90, 3.15)	2.97 (2.80, 3.15)
# of FQHCs per 1000 persons					
	0.02 (0.02, 0.02)	0.02 (0.02, 0.02)	0.02 (0.02, 0.02)	0.02 (0.02, 0.02)	0.02 (0.02, 0.02)
# of RHCs per 1000 persons				*	
	0.01 (0.01, 0.01)	0.01 (0.01, 0.01)	0.01 (0.00, 0.01)	0.01 (0.00, 0.01)	0.01 (0.01, 0.01)

Abbreviations: CRC, Colorectal Cancer; FQHC, Federally Qualified Health Centers; MCO, Managed Care Organizations; PCP, Primary Care Physicians; RHC, Rural Health Centers.

*Note*: Percentage of each individual‐level factor is presented. Estimates for county‐level factors include 95% confidence interval are presented. All estimates were weighted by a composite inverse probability weighting score based on an enrollee's propensity to enroll longer in Medicaid and to use primary care services during the study period. Table S8 presents weighted individual‐ and county‐level characteristics of overall study population and enrollees who had standard colonoscopy. Distributions of each factor in Columns (2)–(4) are compared to distributions of each factor in Column (1). Any CRC tests include any stool‐based tests (gFOBT, FIT, or FIT‐DNA) or any visual tests (standard colonoscopy, flexible sigmoidoscopy, or CT colonography). Enrollees who received any CRC tests include 393 enrollees who received both stool‐based tests and visual tests during the study period.

* *p* < 0.05

** *p* < 0.01

*** *p* < 0.001.

Comparing by testing modality (Table 1, Columns 3 and 4) versus no CRC tests, we generally observed similar patterns as when we compared those with any CRC tests versus those with no CRC tests. However, we observed a larger proportion of enrollees residing in metro counties >1 million population among those who had stool‐based tests alone than those with no CRC tests (65.6% vs. 52.7%, *p*‐value<0.01) but a smaller proportion (46.0% vs. 52.7%, *p*‐value<0.01) when comparing enrollees who had visual tests alone to those who had no CRC tests. In addition, more enrollees enrolled for >360 days per calendar year among those with visual tests alone versus those with no CRC tests (45.0% vs. 37.2%, *p*‐value<0.01). Fewer enrollees utilized no primary care services (20.8% vs. 54.3%, *p*‐value<0.001) among those with stool‐based tests alone versus those with no CRC tests. More enrollees used primary care services 1–2 times per calendar year among those who had stool‐based tests alone (50.6% vs. 19.5%, *p*‐value<0.001). County‐level median household income was lower among those who had visual tests alone versus those with no CRC tests ($53,463.13 vs. $54,593.02 *p*‐value<0.05). We observed a smaller percentage of non‐Hispanic White residents per county among those with stool‐based tests alone than those with no CRC tests (68.53% vs. 73.78%, *p*‐value<0.05). A significantly larger percentage of residents under poverty per county was observed among those with visual tests alone versus enrollees with no CRC tests (14.62% vs. 14.45%, *p*‐value<0.05). We observed a significantly larger number of primary care physicians (0.82 vs. 0.78, *p*‐value<0.05) and specialists (2.85 vs. 2.43, *p*‐value<0.001) and hospital beds (3.02 vs. 2.82, *p*‐value<0.01) per 1000 persons per county was observed among those with stool‐based tests alone versus those with no CRC tests.

Table 2 presents weighted odds ratios (ORs) of receiving CRC tests among the study population, adjusting for individual‐level factors (Column 1s) and for individual‐ and area‐level factors (Column 2s). We observed the same significant individual‐level factors associated with receiving any CRC tests when we adjusted for individual‐level factors alone (Column 1) and both individual‐ and county‐level factors (Column 2).

**TABLE 2:** Weighted associations (Odds ratios [95% CI]) between Individual‐ and County‐level characteristics of Medicaid enrollees and receipt of CRC tests.

	Any CRC tests		Stool‐based tests alone		Visual tests alone	
	(1)	(2)	(1)	(2)	(1)	(2)
Individual‐level characteristics						
Age						
50–54	REF	REF	REF	REF	REF	REF
55–59	1.09 [0.90, 1.31]	1.07 [0.89, 1.29]	1.16 [0.83, 1.62]	1.15 [0.82, 1.61]	1.06 [0.86, 1.30]	1.04 [0.85, 1.27]
60–64	0.70** [0.52, 0.96]	0.70** [0.52, 0.93]	0.64** [0.43, 0.96]	0.64** [0.43, 0.96]	0.68*** [0.50, 0.91]	0.67*** [0.51, 0.90]
Male	1.47*** [1.23, 1.76]	1.49*** [1.25, 1.77]	1.51*** [1.14, 2.00]	1.54*** [1.16, 2.05]	1.41*** [1.16, 1.70]	1.42*** [1.18, 1.72]
Race/Ethnicity						
Non‐Hispanic White	REF	REF	REF	REF	REF	REF
Non‐Hispanic Black	1.16 [0.91, 1.48]	1.08 [0.82, 1.41]	0.64* [0.40, 1.02]	0.56** [0.35, 0.91]	1.25* [0.96, 1.63]	1.17 [0.87, 1.57]
Hispanic	1.57** [1.09, 2.25]	1.57** [1.09, 2.26]	1.21 [0.69, 2.13]	1.07 [0.61, 1.86]	1.67*** [1.13, 2.45]	1.69*** [1.14, 2.51]
Other	1.63*** [1.18, 2.27]	1.57*** [1.13, 2.17]	1.66** [1.06, 2.58]	1.54** [1.00, 2.38]	1.59** [1.11, 2.26]	1.53** [1.07, 2.20]
Rurality of residence						
Metro counties >1 million population	REF	REF	REF	REF	REF	REF
Metro counties <1 million population	0.91 [0.61, 1.35]	0.99 [0.58, 1.68]	0.94 [0.55, 1.60]	1.14 [0.55, 2.36]	0.92 [0.60, 1.41]	0.89 [0.50, 1.57]
Nonmetro counties	0.84 [0.58, 1.22]	0.77 [0.43, 1.36]	1.05 [0.59, 1.89]	2.95** [1.29, 6.73]	0.85 [0.57, 1.29]	0.67 [0.36, 1.25]
Additional eligibility criteria						
Chronically Ill	0.23* [0.04, 1.27]	0.21* [0.04, 1.17]	—	—	0.29 [0.05, 1.73]	0.26 [0.04, 1.55]
Total number of chronic conditions						
0	REF	REF	REF	REF	REF	REF
1–2	2.15*** [1.68, 2.75]	2.13*** [1.67, 2.72]	1.66** [1.07, 2.56]	1.66** [1.08, 2.56]	2.37*** [1.82, 3.08]	2.35*** [1.81, 3.05]
3–4	3.19*** [2.31, 4.40]	3.21*** [2.34, 4.41]	2.50*** [1.54, 4.07]	2.50*** [1.52, 4.10]	3.60*** [2.57, 5.04]	3.64*** [2.61, 5.07]
5+	3.54*** [2.53, 4.95]	3.52*** [2.54, 4.88]	1.97*** [1.29, 3.01]	2.02*** [1.33, 3.09]	3.98*** [2.78, 5.72]	3.94*** [2.77, 5.61]
Missing	0.34*** [0.28, 0.42]	0.34*** [0.28, 0.41]	0.54*** [0.37, 0.79]	0.55*** [0.38, 0.79]	0.34*** [0.27, 0.42]	0.33*** [0.27, 0.41]
Frequency of primary care services use per calendar year						
0	REF	REF	REF	REF	REF	REF
1–2	3.92*** [3.16, 4.87]	3.96*** [3.21, 4.89]	6.57*** [3.89, 11.10]	6.57*** [3.88, 11.11]	3.61*** [2.86, 4.55]	3.63*** [2.88, 4.57]
2–4	1.36** [1.04, 1.77]	1.38** [1.06, 1.79]	2.71*** [1.49, 4.91]	2.78*** [1.54, 5.03]	1.26 [0.95, 1.69]	1.27 [0.95, 1.70]
5+	0.57*** [0.42, 0.77]	0.57*** [0.43, 0.77]	1.25 [0.66, 2.36]	1.26 [0.66, 2.38]	0.53*** [0.38, 0.74]	0.53*** [0.38, 0.74]
County‐level characteristics						
Median household income		1.00* [1.00, 1.00]		1.00 [1.00, 1.00]		1.00 [1.00, 1.00]
Percent Non‐Hispanic White		1.00 [0.98, 1.03]		0.95*** [0.92, 0.98]		1.02 [0.99, 1.04]
Percent unemployed among population of 16+ years		0.74** [0.58, 0.95]		0.88 [0.58, 1.34]		0.78* [0.60, 1.0]

*Note*: All estimates were weighted by a composite inverse probability weighting score based on an enrollee's propensity to enroll longer in Medicaid and to use primary care services during the study period and adjusted for all individual‐ and county‐level characteristics. Table S9 presents weighted associations between individual‐ and county‐level characteristics and receipt of standard colonoscopy. Column (1) adjusts for individual‐level characteristics only; Column (2) adjusts for both individual‐ and county‐level characteristics.

Abbreviations: CRC, Colorectal Cancer; FQHC, Federally Qualified Health Centers; MCO, Managed Care Organizations; PCP, Primary Care Physicians; RHC, Rural Health Centers.

* *p* < 0.05.

** *p* < 0.01.

*** *p* < 0.001.

Significant individual‐level factors associated with an increased likelihood of receiving any CRC tests include being male (*p*‐value<0.001), being Hispanic (*p*‐value<0.01), or of other racial/ethnic groups (*p*‐value<0.001), having at least one chronic condition (*p*‐value<0.001), and using primary care services ≤4 times per calendar year (*p*‐value<0.01). The adjusted odds of receiving any CRC tests (Column 2) seemed to increase with the total number of chronic conditions: enrollees with 1–2 conditions have 2.13 times (95% CI 1.67–2.72, *p*‐value<0.001) those of the enrollees with no chronic conditions; enrollees with 3–4 conditions, 3.21 times (95% CI 2.34–4.41, *p*‐value<0.001); and enrollees with 5+ conditions, 3.52 times (95% CI 2.54–4.88, *p*‐value<0.001). In addition, we observed that using primary care services 1–2 times per calendar year (OR 3.96; 95% CI 3.21, 4.89, *p*‐value<0.001) showed a stronger association between receipt of any CRC tests than using primary care services 2–4 times per calendar year (OR 1.38; 95% CI 1.06, 1.79, *p*‐value<0.01) (Table 2, *Any CRC Tests* Column 2). Significant individual‐level factors associated with a decreased likelihood of receiving any CRC tests include being 60–64 years at enrollment (*p*‐value<0.01), having chronically ill as an additional eligibility criterion (*p*‐value<0.05), and using primary care services >4 times per calendar year (*p*‐value<0.001) (Table 2, *Any CRC Tests* Columns 1 and 2). Finally, we observed that a decreased likelihood of receiving any CRC tests was associated with an increase in unemployment percent per county (OR 0.74; 95% CI 0.58–0.96, *p*‐value<0.01, Table 2, *Any CRC Tests* Column 2).

Significant factors associated with stool‐based tests alone include being male (*p*‐value<0.001), being of other racial/ethnic groups (*p*‐value<0.01), having ≥1 chronic condition (*p*‐value<0.01), and using primary care services 1–4 times per calendar year (*p*‐value<0.001) (Table 2, *Stool‐based Tests Alone* Columns 1 and 2). When adjusting for county‐level characteristics (Table 2, *Stool‐based Tests Alone* Column 2), we observe that residing in non‐metro counties becomes significantly associated with an increased likelihood of receiving stool‐based tests (OR 2.95; 95% CI 1.29–6.73, *p*‐value<0.01). Significant individual‐level factors associated with a decreased likelihood of receiving stool‐based tests include being 60–64 years at enrollment (*p*‐value<0.01) and being non‐Hispanic Black (*p*‐value<0.05 for Table 2, *Stool‐based Tests Alone* Column 1 and *p*‐value<0.01 for *Stool‐based Tests Alone* Column 2). In addition, an increase in county‐level percent non‐Hispanic White (*p*‐value<0.001) was significantly associated with a reduced likelihood of an enrollee's receipt of stool‐based tests alone.

Significant factors associated with receiving CRC visual tests alone include being male (*p*‐value<0.001), being Hispanic (*p*‐value<0.001) or of other racial/ethnic groups (*p*‐value<0.001), having at least one chronic condition (*p*‐value<0.001), and using primary care services ≤2 times per calendar year (*p*‐value<0.001) (Table 2, *Visual Tests Alone* Columns 1 and 2). When we adjusted for individual‐level factors only (Table 2, *Visual Tests Alone* Column 1), being non‐Hispanic Black was significantly associated with an increased likelihood of receiving visual tests alone (OR 1.25, 95% CI 0.96, 1.63, *p*‐value<0.05), suggesting that being non‐Hispanic White is significantly associated with receiving visual tests alone. Significant factors associated with a decreased likelihood of receiving visual tests include being 60–64 years at enrollment (*p*‐value<0.001) and using primary care services >5 times per calendar year (*p*‐value<0.001). In addition, an increase in county‐level percent unemployed was significantly associated with a decreased likelihood of receiving visual tests alone (OR 0.78; 95% CI 0.60–1.00, *p*‐value<0.05). Table S11 presents unweighted associations between the study population's individual‐ and county‐level characteristics with receipt of CRC testing.

## 4 DISCUSSION

Using Pennsylvania Medicaid administrative claims data from 2015 to 2019, we estimated the proportions of individuals who were newly enrolled continuously in Medicaid and have received CRC testing services and examined individual‐ and area‐level factors associated with CRC testing in the post‐ACA expansion. Population‐based surveys or Medicare claims data have been the primary sources describing CRC screening patterns in the U.S.^17^, ^19^, ^38^, ^39^; only a handful of studies have explored CRC screening among age‐eligible Medicaid enrollees. Using Medicaid administrative claims data from one of the largest Medicaid programs in the U.S., we provide new evidence regarding patterns of CRC testing services use and a more comprehensive understanding of significant factors associated with CRC testing among vulnerable population groups whose sociodemographic and environmental characteristics may result in lower rates of CRC testing.

Our findings of significant predictors of CRC testing among newly enrolled Medicaid enrollees in Pennsylvania are similar to those found in Oregon, the only other state with published research on CRC screening use among a newly Medicaid‐enrolled population. Having ≥1 chronic condition, living in an urban area, and having ≥4 primary care visits in the year after turning 50 years were significant facilitators for CRC screening in Oregon.^40^, ^41^ The discrepancy in results regarding primary care visits between our findings and the findings from Oregon may be attributed to different study populations, as the Oregon study may include a younger cohort than our study cohort.

One of the significant factors associated with receipt of CRC testing–both stool‐based and visual tests–in our study was PCP services use. As physicians' recommendations are one of the strongest facilitators for receipt of CRC screening services, any interactions with physicians will likely only increase the likelihood that enrollees are screened relative to if they did not have access to healthcare.^42^ As CRC testing services require either an order from a PCP for stool‐based testing or a referral to a gastroenterologist by a PCP for visual testing, a longer enrollment period in Medicaid may allow enrollees to establish care and receive preventive services. Many studies confirm that the ACA Medicaid expansion effectively lowered the number of uninsured individuals and thus facilitated access to healthcare. We observe that the longer the enrollment period, the larger the proportion of the study population that received stool tests. In 2015, about 2.6% of the study population received any stool‐based tests, which increased to 4.1% in 2019 (full data not shown). However, this pattern was less apparent with visual tests as they require a longer time interval (8.9% in 2015 and 6.2% in 2019). In addition, the finding that using primary care services ≤2 times per year and having a chronic condition are strong predictors of receiving any CRC tests demonstrates the complex decision‐making process between providers and patients when chronic condition management may need to be prioritized relative to preventive care.^43^ It also suggests that future studies should investigate provider‐level factors associated with CRC screening and patients' preferences for cancer preventive services, including CRC screening.

We observed nuanced associations by frequency of PCP services use by screening modality: we observed that 2+ visits to PCP per calendar year were a significant facilitator for stool‐based tests alone and that 5+ visits to PCP per calendar year were significantly associated with a decreased likelihood of receiving visual tests, while adjusting for other individual‐ and area‐level characteristics. This further highlights that more comprehensive understanding of other potentially relevant factors, that is, receipt of other preventive services or severity of chronic conditions, need to be factored in to design more tailored intervention strategies to improve CRC prevention in these vulnerable population groups.

In addition, our study sheds light on the role of some pre‐disposing factors, including sex, race/ethnicity, health insurance, and rurality of residence, on receipt of CRC testing services. We observed a significantly increased likelihood of receiving visual tests among Hispanic individuals who newly enrolled in Medicaid after the Medicaid expansion versus their non‐Hispanic White counterparts. This finding differed from other studies reporting that being Hispanic has been associated with lower CRC screening rates in the general adult population age‐eligible for CRC screening,^44^ which might have been due to a high correlation between the likelihood of being uninsured and being Hispanic. Also, we observed a positive association between being male and receiving any CRC tests among male individuals who newly enrolled in Medicaid after Medicaid expansion than their female counterparts. While there are mixed findings about the associations between sex and receipt of CRC screening,^45^, ^46^, ^47^ our study provides evidence that more targeted interventions may be more effective in reaching out to female Medicaid enrollees to reduce the burden of CRC among Medicaid population. Finally, we observed that residing in non‐metro counties is significantly associated with an increased likelihood of receiving stool‐based tests controlling for other individual‐ and area‐level characteristics. This additional observation underscores the importance of strategies to improve CRC screening that are more racially and geographically tailored.

There are limitations to our study. First, the study data do not allow us to distinguish CRC screening services from surveillance after a previous diagnosis. We addressed this limitation by excluding those with a history of colon/rectal cancer at enrollment or diagnosed with colon/rectal cancer within the first quarter of enrollment, adjusting for the number of chronic conditions at enrollment, and excluding those with multiple visual tests. Second, this study cannot report on guideline‐concordant CRC screening, as Medicaid claims data provide records of CRC testing services only while enrolled in Medicaid. However, we focused on enrollees who newly gained insurance coverage in 2015. Hence, this group may have been more likely to have been previously uninsured and lacked access to health care. Third, Medicaid claims data do not collect information on other relevant factors, including the severity of chronic conditions and provider and/or clinic‐related factors (distance to the nearest endoscopy center, affiliation with large health systems, and a panel size of patients eligible for CRC screening).^8^, ^48^, ^49^, ^50^, ^51^, ^52^ Thus, further research is needed to understand better interactions between individual‐ and provider‐level factors which play a critical role in their decision‐making process for CRC screening.^50^, ^53^, ^54^

Despite these limitations, our study contributes to our understanding about factors associated with CRC prevention from vulnerable population groups who may face more barriers to timely and guideline‐recommended CRC screening. Our findings reported lower CRC screening rates among low‐income non‐elderly adults who newly gained insurance coverage than those published in the literature examining the total population: about 1 in 3 enrollees in our study population received any CRC testing services, which is lower than the national average of 60.5% among all adults aged 50–64 years in 2018.^39^ We also identified unique sets of significant factors associated with CRC testing, which are different by modality and also from other population groups. All in all, our findings suggest the need for further concerted efforts from stakeholders to identify strategies tailored to patients' racial, geographic, and clinical conditions and for future studies in understanding provider‐specific factors associated with CRC screening and the decision‐making processes of providers and patients in receipt of CRC screening services.

## AUTHOR CONTRIBUTIONS

**Kirsten Y. Eom:** Conceptualization (equal); formal analysis (equal); investigation (equal); methodology (equal); project administration (equal); writing – original draft (equal); writing – review and editing (equal). **Scott D. Rothenberger:** Formal analysis (equal); methodology (equal); writing – review and editing (equal). **Marian P. Jarlenski:** Formal analysis (equal); methodology (equal); writing – review and editing (equal). **Robert E. Schoen:** Writing – review and editing (equal). **Evan S. Cole:** Data curation (equal); project administration (equal); writing – review and editing (equal). **Lindsay M. Sabik:** Conceptualization (equal); data curation (equal); formal analysis (equal); methodology (equal); project administration (equal); supervision (equal); writing – review and editing (equal).

## FUNDING INFORMATION

This research received no specific grant from any funding agency in the public, commercial, or not‐for‐profit sectors.

## CONFLICT OF INTEREST STATEMENT

All the listed authors have no conflicts of interest or financial disclosures.

## ETHICS APPROVAL STATEMENT

This study was determined to be exempt from human subjects review by the University of Pittsburgh Institutional Review Board. There were no animals involved in this study.

## Supporting information


Data S1.

## Data Availability

The data underlying this article cannot be shared due to the data use agreement with the Pennsylvania Department of Human Services to protect patients' privacy.
