# Supplementary material for: Enrollee characteristics and receipt of colorectal cancer testing in Pennsylvania after adoption of the Affordable Care Act Medicaid expansion
Source: Cancer Med. 2023 Jun 17;12(14):15455–67. doi: 10.1002/cam4.6168 (PMC10417095; doi:10.1002/cam4.6168)
Supplement: Supplementary file 1 — Data S1. [file CAM4-12-15455-s001.docx]

**Enrollee Characteristics and Receipt of Colorectal Cancer Testing in Pennsylvania After Adoption of the Affordable Care Act Medicaid Expansion**

*Supplemental Document*

[Table S1. U.S. Preventive Services Task Force (USPSTF) Guidelines for Colorectal Cancer Screening 2](#_Toc110605962)

[Table S2. The CMS Chronic Conditions Data Warehouse Condition Algorithms for Colorectal Cancer 4](#_Toc110605963)

[Table S3. Billing Codes for Colorectal Cancer Testing Services 5](#_Toc110605964)

[Table S4. The CMS Chronic Conditions Data Warehouse (CCW) 6](#_Toc110605965)

[Table S5. Current Procedural Terminology (CPT) codes for primary care services 8](#_Toc110605966)

[Table S6. Provider specialty codes in Medicaid outpatient claims files 12](#_Toc110605967)

[Calculating Inverse Probability Censoring Weights (IPCW) for Continuous Enrollment from 2015 to 2019 13](#_Toc110605968)

[Table S7. Baseline Characteristics of Newly Expansion Medicaid Enrollees in 2015 by Continuous Enrollment Status 13](#_Toc110605969)

[Figure S1. Absolute Standardized Differences between Unweighted and Weighted Means for All Covariates for IPCW 19](#_Toc110605970)

[Figure S2. Unweighted, Weighted Proportions, and the Differences of Age among Newly Enrolled Medicaid Enrollees by Continuous Enrollment from 2015 to 2019 20](#_Toc110605971)

[Figure S3. Unweighted, Weighted Proportions, and the Differences of Sex among Newly Enrolled Medicaid Enrollees by Continuous Enrollment from 2015 to 2019 21](#_Toc110605972)

[Figure S4. Unweighted, Weighted Proportions, and the Differences of Race/ethnicity Newly Enrolled Medicaid Enrollees by Continuous Enrollment from 2015 to 2019 22](#_Toc110605973)

[Figure S5. Unweighted, Weighted Proportions, and the Differences of Additional Basis of Eligibility Newly Enrolled Medicaid Enrollees by Continuous Enrollment from 2015 to 2019 23](#_Toc110605974)

[Figure S6. Unweighted, Weighted Proportions, and the Differences of Managed Care Organization (MCO) Region Newly Enrolled Medicaid Enrollees by Continuous Enrollment from 2015 to 2019 24](#_Toc110605975)

[Figure S7. Unweighted, Weighted Proportions, and the Differences of Total Number of Chronic Conditions at Enrollment Newly Enrolled Medicaid Enrollees by Continuous Enrollment from 2015 to 2019 25](#_Toc110605976)

[Figure S8. Unweighted, Weighted Proportions, and the Differences of Mean Number of Enrolled Days in 2015 Newly Enrolled Medicaid Enrollees by Continuous Enrollment from 2015 to 2019 26](#_Toc110605977)

[Figure S9. Unweighted, Weighted Proportions, and the Differences of Rurality of Residence at Enrollment Newly Enrolled Medicaid Enrollees by Continuous Enrollment from 2015 to 2019 27](#_Toc110605978)

[Calculating Inverse Probability of Using Any Primary Care Services (IPTW) from 2015 to 2019 28](#_Toc110605979)

[Table S8. Baseline Characteristics of Newly expansion Medicaid Enrollees in 2015 by Primary Care Services Use from 2015 to 2019 28](#_Toc110605980)

[Figure S10. Absolute Standardized Differences between Unweighted and Weighted Means for All Covariates for IPTW 34](#_Toc110605981)

[Figure S11. Unweighted, Weighted Proportions, and the Differences of Age among Newly Continuously Enrolled Medicaid Enrollees by Primary Care Services Use from 2015 to 2019 35](#_Toc110605982)

[Figure S12. Unweighted, Weighted Proportions, and the Differences of Sex among Newly Continuously Enrolled Medicaid Enrollees by Primary Care Services Use from 2015 to 2019 36](#_Toc110605983)

[Figure S13. Unweighted, Weighted Proportions, and the Differences of Race/Ethnicity among Newly Continuously Enrolled Medicaid Enrollees by Primary Care Services Use from 2015 to 2019 37](#_Toc110605984)

[Figure S14. Unweighted, Weighted Proportions, and the Differences of Additional Basis of Eligibility among Newly Continuously Enrolled Medicaid Enrollees by Primary Care Services Use from 2015 to 2019 38](#_Toc110605985)

[Figure S15. Unweighted, Weighted Proportions, and the Differences of Managed Care Organization (MCO) Region among Newly Continuously Enrolled Medicaid Enrollees by Primary Care Services Use from 2015 to 2019 39](#_Toc110605986)

[Figure S16. Unweighted, Weighted Proportions, and the Differences of Total Number of Chronic Conditions at Enrollment among Newly Continuously Enrolled Medicaid Enrollees by Primary Care Services Use from 2015 to 2019 40](#_Toc110605987)

[Figure S17. Unweighted, Weighted Proportions, and the Differences of Total Enrollment Length per Calendar Year (Days) among Newly Continuously Enrolled Medicaid Enrollees by Primary Care Services Use from 2015 to 2019 41](#_Toc110605988)

[Figure S18. Unweighted, Weighted Proportions, and the Differences of Rurality of Residence at Enrollment among Newly Continuously Enrolled Medicaid Enrollees by Primary Care Services Use from 2015 to 2019 42](#_Toc110605989)

[Figure S19. Distribution of the Composite Inverse Probability Study Weights (IPSW) based on Inverse Probability Censoring Weights (IPCWs) and Inverse Probability Treatment Weights (IPTWs) 43](#_Toc110605990)

[Table S9. Unweighted Individual- and County-level Characteristics of Newly Enrolled Expansion Population among Medicaid Enrollees in Pennsylvania 44](#_Toc110605991)

[Table S10. Weighted Individual- and County-level Characteristics of Newly Enrolled Expansion Population among Medicaid Enrollees in Pennsylvania 48](#_Toc110605992)

[Table S11. Unweighted Association (Odds Ratio [95% CI]) between Individual- and County-level Characteristics of Medicaid Enrollees and Receipt of CRC Tests 52](#_Toc110605993)

[Table S12. Weighted Associations (Odds Ratio [95% CI]) between Individual- and County-level Characteristics of Medicaid Enrollees and Receipt of CRC tests 56](#_Toc110605994)

# Table S1. U.S. Preventive Services Task Force (USPSTF) Guidelines for Colorectal Cancer Screening

| **Year** | **Population** | **Recommendations** | | **Grade for Evidence** |
| --- | --- | --- | --- | --- |
|  |  | **Screening Tests** | **Screening Intervals** |  |
| 2002 | Men and women 50+ years old | Screen with periodic FOBT | FOBT: every year | A |
| 2008 | Adults aged 50-75 years | Screen with high-sensitivity FOBT, sigmoidoscopy, or colonoscopy | High-sensitivity FOBT: every year  Sigmoidoscopy: every 5 years, with high-sensitivity FOBT: every 3 years  Standard colonoscopy: every 10 years | A |
|  | Adults aged 76-85 years | Do not screen routinely |  | C |
|  | Adults aged 85+ years | Do not screen |  | D |
|  |  | For all populations, evidence is insufficient to assess the benefits and harms of screening with CT colonography and FIT-DNA. |  | I |
| 2016 | Adults aged 50-75 years | Screen with stool-based tests (gFOBT, FIT, or FIT-DNA), direct visualization tests (flexible sigmoidoscopy, alone or combined with FIT; colonoscopy; and CT colonography), and serology tests (SEPT9 DNA test).  *The USPSTF found no head-to-head studies demonstrating that any of these screening strategies are more effective than others, although they have varying levels of evidence supporting their effectiveness, as well as different strengths and limitations.* | gFOBT: every year  FIT: every year  FIT-DNA: every year or three years  Flexible sigmoidoscopy: every 5 years  Flexible sigmoidoscopy with FIT: flexible sigmoidoscopy every 10 years with FIT every year  Colonoscopy: every 10 years  CT colonography: every 5 years | A |
|  | Adults aged 76-85 years | The decision to screen for colorectal cancer is an individual one. |  | C |
| **FOBT=**Fecal Occult Blood Test; **gFOBT**=guaiac Fecal Occult Blood Test; **FIT**=Fecal Immunochemical Test; **CT**=Computed Tomography  **NOTE** The USPSTF assigns a letter to grade quality of evidence.  **A**: The USPSTF recommends the service. There is high certainty that the net benefit is substantial.  **B:** The USPSTF recommends the service. There is high certainty that the net benefit is moderate or there is moderate certainty that the net benefit is moderate to substantial.  **C**: The USPSTF recommends against routinely providing the service. There may be considerations that support providing the service in an individual patient. There is moderate or high certainty that the net benefit is small.  **D**: The USPSTF recommends against the service. There is moderate or high certainty that the service has no net benefit or that the harms outweigh the benefits.  **I**: The USPSTF concludes that the current evidence is insufficient to assess the balance of benefits and harms of the service. Evidence is lacking, of poor quality, or conflicting, and the balance of benefits and harms cannot be determined. | | | | |

# Table S2. The CMS Chronic Conditions Data Warehouse Condition Algorithms for Colorectal Cancer

| **CCW**  **Algorithm** | **Reference Period**  (# of years) | **Valid ICD-9 / CPT4 / HCPCS Codes^a^** | **Valid ICD-10 / CPT4 / HCPCS Codes^a^** | **Number / Type of Claims to Qualify^b^** |
| --- | --- | --- | --- | --- |
| Colorectal Cancer | 1 year | DX 153.0, 153.1, 153.2, 153.3, 153.4, 153.5, 153.6, 153.7, 153.8, 153.9, 154.0, 154.1, 230.3, 230.4, V10.05, V10.06  **(any diagnosis on the claim)** | DX C18.0, C18.1, C18.2, C18.3, C18.4, C18.5, C18.6, C18.7, C18.8, C18.9, C19, C20, D01.0, D01.1, D01.2, Z85.038, Z85.048  **(any diagnosis on the claim)** | At least 1 inpatient, SNF **OR** 2 HOP or Carrier claims with diagnosis codes |
| **CMS**=Centers for Medicare and Medicaid Services; **CCW**=Chronic Conditions Data Warehouse; **CPT**=Current Procedural Terminology; **HCPCS**=Healthcare Common Procedure Coding System; **SNF**=skilled nursing facility; **HOP**=hospital outpatient.  ^a^ICD-10 codes are effective 10/2015; effective dates for ICD-9 codes vary but are valid through 09/2015.  ^b^Carrier claims refer to claim types 71 and 72 (not DME claim types 81 or 82) and exclude any claims for which line item Berenson-Eggers Type of Service [BETOS] code variable equals D1A, D1B, D1C, D1D, D1E, D1F, D1G (which is DME), or O1A (which is ambulance services). The algorithm intends to exclude claims where the services do not require a licensed health care professional. When 2 claims are required, they must occur at least one day apart. | | | | |

# Table S3. Billing Codes for Colorectal Cancer Testing Services

| **Testing Services** | **CPT Codes** | **HCPCS Codes** |
| --- | --- | --- |
| guaiac FOBT (gFOBT) | 82270 | G0328 |
| Fecal immunochemical (or immunohistochemical) test (FIT, also known as iFOBT) | 82274 | - |
| Stool DNA test (FIT-DNA): Cologuard | 81528 | G0464 |
| Flexible Sigmoidoscopy | 45330-45335; 45337-45338; 45340-45342; 45346-45347; 45349-45350 | G0104 |
| Standard (or optical) Colonoscopy | 44401-44408; 45388-45394; 44397; 45355; 45378-45386; 45388-45389; 45390-45393; 45398 | G0105  G0121 |
| Virtual Colonoscopy or Computed Tomographic (CT) Colonography | 74261-74263 | - |
| **CPT**=Current Procedural Terminology; **HCPCS**=Healthcare Common Procedure Coding System | | |

# Table S4. The CMS Chronic Conditions Data Warehouse (CCW)

| **Common Chronic Conditions** | **Other Chronic or Potentially Disabling Conditions** |
| --- | --- |
| Acute Hypothyroidism | ADHD, Conduct Disorders, and Hyperkinetic Syndrome |
| Acute Myocardial Infarction | Alcohol Use Disorders |
| Alzheimer’s Disease | Anxiety Disorders |
| Alzheimer’s Disease, Related Disorders, or Senile Dementia | Autism Spectrum Disorders |
| Anemia | Bipolar Disorder |
| Asthma | Cerebral Palsy |
| Atrial Fibrillation | Cystic Fibrosis and Other Metabolic Developmental Disorders |
| Benign Prostatic Hyperplasia | Depressive Disorders |
| Cancer, Colorectal | Drug Use Disorders |
| Cancer, Endometrial | Epilepsy |
| Cancer, Breast | Fibromyalgia, Chronic Pain and Fatigue |
| Cancer, Lung | Human Immunodeficiency Virus and/or Acquired Immunodeficiency Syndrome (HIV/AIDS)* |
| Cancer, Prostate | Intellectual Disabilities and Related Conditions |
| Cataract | Learning Disabilities |
| Chronic Kidney Disease | Leukemias and Lymphomas |
| Chronic Obstructive Pulmonary Disease | Liver Disease, Cirrhosis and Other Liver Conditions (except Viral Hepatitis) |
| Depression | Migraine and Chronic Headache |
| Diabetes | Mobility Impairments |
| Glaucoma | Multiple Sclerosis and Transverse Myelitis |
| Heart Failure | Muscular Dystrophy |
| Hip/Pelvic Fracture | Obesity |
| Hyperlipidemia | Opioid Use Disorder (OUD) |
| Hypertension | Other Developmental Delays |
| Ischemic Heart Disease | Peripheral Vascular Disease (PVD) |
| Osteoporosis | Personality Disorders |
| Rheumatoid Arthritis/Osteoarthritis | Post-Traumatic Stress Disorders (PTSD) |
| Stroke/Transient Ischemic Attack | Pressure and Chronic Ulcers |
|  | Schizophrenia |
|  | Schizophrenia and Other Psychotic Disorders |
|  | Sensory – Blindness and Visual Impairment |
|  | Sensory – Deafness and Hearing Impairment |
|  | Sickle Cell Disease |
|  | Spina Bifida and Other Congenital Anomalies of the Nervous System |
|  | Spinal Cord Injury |
|  | Tobacco Use |
|  | Traumatic Brain Injury and Nonpsychotic Mental Disorders due to Brain Damage |
|  | Viral Hepatitis (General), including:   - Hepatitis A - Hepatitis B (acute or unspecified) - Hepatitis B (chronic) - Hepatitis C (acute) Hepatitis C (chronic) - Hepatitis C (unspecified0 - Hepatitis D - Hepatitis E |
| *There are also variables that measure care (largely Medicaid services) provided to individuals with HIV/AIDS. These variables are not included in the Master Beneficiary Summary File (MBSF) or Medicaid Enrollee Supplemental File (MESF). | |

# Table S5. Current Procedural Terminology (CPT) codes for primary care services

| **CPT Code** | **Description** |
| --- | --- |
| 99201 | Office or other outpatient visit for the evaluation and management of a new patient, which requires these three key components: a problem focused history; a problem focused examination; and straightforward medical decision making. Counseling and/or coordination of care with other physicians, other qualified health care professionals, or agencies are provided consistent with the nature of the problem(s) and the patient's and/or family's needs. Usually, the presenting problem(s) are self-limited or minor. Typically, 10 minutes are spent face-to-face with the patient and/or family. |
| 99202 | Office or other outpatient visit for the evaluation and management of a new patient, which requires these three key components: an expanded problem focused history; an expanded problem focused examination; and straightforward medical decision making. Counseling and/or coordination of care with other physicians, other qualified health care professionals, or agencies are provided consistent with the nature of the problem(s) and the patient's and/or family's needs. Usually, the presenting problem(s) are of low to moderate severity. Typically, 20 minutes are spent face-to-face with the patient and/or family. |
| 99203 | Office or other outpatient visit for the evaluation and management of a new patient, which requires these three key components: a detailed history; a detailed examination; and medical decision making of low complexity. Counseling and/or coordination of care with other physicians, other qualified health care professionals, or agencies are provided consistent with the nature of the problem(s) and the patient's and/or family's needs. Usually, the presenting problem(s) are of moderate severity. Typically, 30 minutes are spent face-to-face with the patient and/or family. |
| 99204 | Office or other outpatient visit for the evaluation and management of a new patient, which requires these three key components: a comprehensive history; a comprehensive examination; and medical decision making of moderate complexity. Counseling and/or coordination of care with other physicians, other qualified health care professionals, or agencies are provided consistent with the nature of the problem(s) and the patient's and/or family's needs. Usually, the presenting problem(s) are of moderate to high severity. Typically, 45 minutes are spent face-to-face with the patient and/or family. |
| 99205 | Office or other outpatient visit for the evaluation and management of a new patient, which requires these three key components: a comprehensive history; a comprehensive examination; and medical decision making of high complexity. Counseling and/or coordination of care with other physicians, other qualified health care professionals, or agencies are provided consistent with the nature of the problem(s) and the patient's and/or family's needs. Usually, the presenting problem(s) are of moderate to high severity. Typically, 60 minutes are spent face-to-face with the patient and/or family. |
| 99211 | Office or other outpatient visit for the evaluation and management of an established patient, that may not require the presence of a physician or other qualified health care professional. Usually, the presenting problem(s) are minimal. Typically, 5 minutes are spent performing or supervising these services. |
| 99212 | Office or other outpatient visit for the evaluation and management of an established patient, which requires at least two of these three key components: a problem focused history; a problem focused examination; straightforward medical decision making. Counseling and/or coordination of care with other physicians, other qualified health care professionals, or agencies are provided consistent with the nature of the problem(s) and the patient's and/or family's needs. Usually, the presenting Problem(s) are self -limited or minor. Typically, 10 minutes are spent face-to-face with the patient and/or family. |
| 99213 | Office or other outpatient visit for the evaluation and management of an established patient, which requires at least two of these three key components: an expanded problem focused history; an expanded problem focused examination; medical decision making of low complexity. Counseling and/or coordination of care with other physicians, other qualified health care professionals, or agencies are provided consistent with the nature of the problem(s) and the patient's and/or family's needs. Usually, the presenting problem(s) are of low to moderate severity. Typically, 15 minutes are spent face-to-face with the patient and/or family. |
| 99214 | Office or other outpatient visit for the evaluation and management of an established patient, which requires at least two of these three key components: a detailed history; a detailed examination; medical decision making of moderate complexity. Counseling and/or coordination of care with other physicians, other qualified health care professionals, or agencies are provided consistent with the nature of the problem(s) and the patient's and/or family's needs. Usually, the presenting problem(s) are of moderate to high severity. Typically, 25 minutes are spent face-to-face with the patient and/or family. |
| 99215 | Office or other outpatient visit for the evaluation and management of an established patient, which requires at least two of these three key components: a comprehensive history; a comprehensive examination; medical decision making of high complexity. Counseling and/or coordination of care with other physicians, other qualified health care professionals, or agencies are provided consistent with the nature of the problem(s) and the patient's and/or family's needs. Usually, the presenting problem(s) are of moderate to high severity. Typically, 40 minutes are spent face-to-face with the patient and/or family. |
| 99386 | Initial comprehensive preventive medicine evaluation and management of an individual including an age and gender appropriate history, examination, counseling/anticipatory guidance/risk factor reduction interventions, and the ordering of laboratory/diagnostic procedures, new patient. |
| 99396 | Periodic comprehensive preventive medicine reevaluation and management of an individual including an age and gender appropriate history, examination, counseling/anticipatory guidance/risk factor reduction interventions, and the ordering of laboratory/diagnostic procedures, established patient; 40-64 years |
| 99499 | Should be limited to cases where there is no other specific E/M code payable by Medicare that describes that service. Reporting CPT code 99499 requires submission of medical records and contractor manual medical review of the service prior to payment. Contractors shall expect reporting under these circumstances to be unusual. |
| 99241 | Office consultation for a new or established patient, which requires these 3 key components: A problem focused history; A problem focused examination; and Straightforward medical decision making. Counseling and/or coordination of care with other providers or agencies are provided consistent with the nature of the problem(s) and the patient's and/or family's needs. Usually, the presenting problem(s) are self-limited or minor. Typically, 15 minutes are spent face-to-face with the patient and/or family. |
| 99242 | Office consultation for a new or established patient, which requires these 3 key components: An expanded problem focused history; An expanded problem focused examination; and Straightforward medical decision making. Counseling and/or coordination of care with other providers or agencies are provided consistent with the nature of the problem(s) and the patient's and/or family's needs. Usually, the presenting problem(s) are of low severity. Typically, 30 minutes are spent face-to-face with the patient and/or family. |
| 99243 | Office consultation for a new or established patient, which requires these 3 key components: A detailed history; A detailed examination; and Medical decision making of low complexity. Counseling and/or coordination of care with other providers or agencies are provided consistent with the nature of the problem(s) and the patient's and/or family's needs. Usually, the presenting problem(s) are of moderate severity. Typically, 40 minutes are spent face-to-face with the patient and/or family. |
| 99244 | Office consultation for a new or established patient, which requires these 3 key components: A comprehensive history; A comprehensive examination; and Medical decision making of moderate complexity. Counseling and/or coordination of care with other providers or agencies are provided consistent with the nature of the problem(s) and the patient's and/or family's needs. Usually, the presenting problem(s) are of moderate to high severity. Typically, 60 minutes are spent face-to-face with the patient and/or family. |
| 99245 | Office consultation for a new or established patient, which requires these 3 key components: A comprehensive history; A comprehensive examination; and Medical decision making of high complexity. Counseling and/or coordination of care with other physicians, other qualified health care professionals, or agencies are provided consistent with the nature of the problem(s) and the patient's and/or family's needs. Usually, the presenting problem(s) are of moderate to high severity. Typically, 80 minutes are spent face-to-face with the patient and/or family. |
| G0402 | Initial Preventive Physical Examination: face-to-face visit, services limited to a new patient during the first 12 months of Medicare enrollment. |
| G0438 | Annual wellness visit; includes a personalized prevention plan of service (PPS), initial visit |
| G0439 | Annual wellness visit; includes a personalized prevention plan of service (PPS), subsequent visit |
| T1015 | All-inclusive clinic visit, which includes the medical diagnosis and treatment services rendered at a federally qualified health center or community health center. Only federally qualified health centers (FQHC) or community health centers (CHC) can file claims with HCPCS code T1015. |
| U4 | Medicaid level of care 4, as defined by each state |
| U5 | Medicaid level of care 5, as defined by each state |
| U7 | Medicaid level of care 7, as defined by each state |
| U8 | Medicaid level of care 8, as defined by each state |

# Table S6. Provider specialty codes in Medicaid outpatient claims files

| Provider specialty codes | Description |
| --- | --- |
| 08 | Clinic |
| 31 | Physician |
| 80 | Federally Qualified Health Centers (FQHCs) |
| 81 | Rural Health Clinics (RHCs) |
| 83 | Family planning clinic |
| 92 | Family Health |
| 93 | Nurse Practitioner (Primary care) |
| 100 | Physician Assistant |
| 316 | Family Practice |
| 318 | General Practitioner |
| 322 | Internal Medicine |
| 344 | General Internist |

# Calculating Inverse Probability Censoring Weights (IPCW) for Continuous Enrollment from 2015 to 2019

We compared sociodemographic characteristics of 62,593 enrollees who meet exclusion criteria 1 – 10 (Figure 1) and do not stay continuously enrolled from 2015 to 2019 to those of 15,547 enrollees of our study population in Appendix Table 6. A significantly larger proportion of enrollees who are not continuously enrolled from 2015 to 2019 is 60-64 years old at enrollment than enrollees remained continuously enrolled in Medicaid (25% vs. 7%). We observe a larger proportion of female enrollees (55% vs. 52%) and of enrollees from other ethnic groups (12% vs. 9%) among those continuously enrolled from 2015 to 2019. Overall, the prevalence of additional basis of eligibility among enrollees who disenrolled from Medicaid at any point from 2015 to 2019 is higher than that of enrollees continuously enrolled. Larger proportions of enrollees who stayed continuously enrolled reside in metro counties with >1 million population or urban or completely rural counties. The number of enrollees within the Lehigh Capital MCO region among enrollees are continuously enrolled in Medicaid is significantly smaller while the number of enrollees within the Southwest CMO region is significantly larger. The mean total length of enrollment in 2015 is significantly shorter among enrollees who stayed enrolled throughout from 2015 to 2019 as the number of enrollees who stayed enrolled in Medicaid for almost the entire calendar year of 2015 is substantially higher among enrollees who did not stay continuously enrolled. The number of enrollees with 5 or more chronic conditions is significantly larger among enrollees who did not remain continuously enrolled in Medicaid; the number of enrollees with missing information for their chronic conditions is larger among enrollees continuously enrolled in Medicaid.

# Table S7. Baseline Characteristics of Newly Expansion Medicaid Enrollees in 2015 by Continuous Enrollment Status

|  | **Not Continuously Enrolled** | **Continuously**  **Enrolled** |
| --- | --- | --- |
| **N** | 62,593 | 15,547 |
| ***%*** | *80.10* | *19.90* |
| **Outcome Measures** |  |  |
| Any CRC testing*** | 9.59 | 8.07 |
| Any stool-based tests^1^ | 1.59 | 1.37 |
| Any visual tests^2^*** | 8.04 | 6.66 |
| Any stool-based and visual tests | 0.25 | 0.26 |
| Standard Colonoscopy*** | 7.93 | 6.63 |
| **Age***** |  |  |
| 50-54 | 41.67 | 47.43 |
| 55-59 | 32.87 | 45.13 |
| 60-64 | 25.46 | 7.44 |
| **Sex***** |  |  |
| Female | 51.55 | 54.80 |
| Male | 48.45 | 45.20 |
| **Race/Ethnicity***** |  |  |
| Non-Hispanic White | 63.59 | 64.43 |
| Non-Hispanic Black | 17.94 | 17.10 |
| Hispanic | 8.99 | 6.29 |
| Other | 9.48 | 12.18 |
| **Additional Basis of eligibility** |  |  |
| Children and Families*** | 6.19 | 2.61 |
| Disabled*** | 7.66 | 2.70 |
| Chronically Ill*** | 0.24 | 0.08 |
| Healthy Horizons*** | 11.36 | 1.54 |
| **Rurality of residence***** |  |  |
| Metro counties of >1 million population | 51.43 | 53.89 |
| Metro counties <1 million population | 37.33 | 32.77 |
| Nonmetro (Urban or completely rural counties) | 11.24 | 13.35 |
| **MCO regions***** |  |  |
| Lehigh Capital | 22.99 | 18.56 |
| New East | 14.60 | 14.68 |
| New West | 6.71 | 7.16 |
| Southeast | 34.13 | 35.83 |
| Southwest | 21.57 | 23.77 |
| **Total enrollment days^[[1]](#footnote-1)^***** |  |  |
| Mean  (95% CI) | 329.47  (329.04, 329.89) | 311.46  (310.58, 312.35) |
| **Total number of chronic conditions***** |  |  |
| 0 | 21.51 | 23.01 |
| 1 – 2 | 23.72 | 21.95 |
| 3 – 4 | 18.57 | 12.21 |
| 5 or more | 22.79 | 8.88 |
| Missing | 13.41 | 33.94 |
| **NOTE** Continuous enrollment is defined as being enrolled in Medicaid for at least 180 days without a gap <15 days in enrollment in a calendar year from 2015 to 2019.  Except for Total enrollment days, percentage of each factor is presented.  ^1^This group excludes beneficiaries who had any visual CRC tests.  ^2^This group excludes beneficiaries who had any stool-based CRC tests  ***p<0.001, **p<0.01, *p<0.05 | | |

To adjust for these differences in these observed characteristics by continuous enrollment status, we implemented the following steps to calculate the inverse probability censoring weight, *IPCW*, for each enrollee in SAS 9.4.

Step 1. Run a multivariable logistic regression model estimating the likelihood of staying continuously enrolled in Medicaid from 2015 to 2019 (Model 1)

$$Y(\Pr\left( {CONTENROLL}_{i}=1 \right))=\beta_{0}+\beta_{1}{Age}_{i}+\beta_{2}{Sex}_{i}+\beta_{3}{RaceEthnicity}_{i}+\beta_{4}{ChildrenandFamilies}_{i}+{\beta_{5}{Disabled}_{i}+\beta_{6}{ChronicallyIll}_{i}+\beta_{7}{HealthyHorizon}_{i}+\beta}_{8}{RuralityResidence}_{i}+\beta_{9}{MCORegion}_{i}+\beta_{10}{TotalEnrollLength}_{i}+\beta_{11}{TotalChronicConditions}_{i}$$

Model 1

In Model 1, *CONTENROLL* is a binary measure of whether an enrollee, *i*, stays enrolled in Medicaid from 2015 to 2019. Except for *TotalEnrollLength*, which is a continuous variable*,* all other covariates are either binary or categorical.

The following sets of outputs provide the various diagnostics tests of Model 1.

| **Model Fit Statistics** | | |
| --- | --- | --- |
| **Criterion** | **Intercept Only** | **Intercept and Covariates** |
| **AIC** | 77,979.786 | 68,571.354 |
| **SC** | 77,989.052 | 68,756.679 |
| **-2 Log L** | 77,977.786 | 68, 531.354 |

Akaike Information Criterion (AIC) and Schwarz Criterion (SC) are used to assess the model fit; AIC and SC penalize the number of predictors in the model. AIC is used for the comparison of non-nested models, and the model with the smallest AIC is desired. Based on the AIC and SC values above, the model with intercept and covariates is preferred.

| **Testing Global Null Hypothesis: BETA=0** | | | |
| --- | --- | --- | --- |
| **Test** | **Chi-Square** | **DF** | **Pr>ChiSq** |
| **Likelihood Ratio** | 9,446.4316 | 19 | <.0001 |
| **Score** | 8,442.6069 | 19 | <.0001 |
| **Wald** | 6,987.8242 | 19 | <.0001 |

The likelihood ratio, score, and Wald tests evaluate the null hypothesis that at least one of the predictors’ regression coefficient is not equal to zero in the model. All three tests reject the null hypothesis; at least one of the predictors’ regression coefficient is not equal to zero in Model 1.

| **Analysis of Maximum Likelihood Estimates** | | | | | | | |
| --- | --- | --- | --- | --- | --- | --- | --- |
| **Parameter** |  | **DF** | **Estimate** | **Standard Error** | **Wald Chi-Square** | **Pr>ChiSq** | **Exp(Est)** |
| **Intercept** |  | 1 | -0.6776 | 0.0559 | 146.9687 | <.0001 | 0.508 |
| **agec** | 0 | 1 | 0.4434 | 0.0148 | 903.0387 | <.0001 | 1.558 |
| **agec** | 1 | 1 | 0.5886 | 0.0149 | 1559.1524 | <.0001 | 1.801 |
| **sex** |  | 1 | 0.2318 | 0.0193 | 144.9762 | <.0001 | 1.261 |
| **race_ethnic** | 1 | 1 | 0.00771 | 0.0171 | 0.2031 | 0.6523 | 1.008 |
| **race_ethnic** | 2 | 1 | -0.0989 | 0.0218 | 20.5428 | <.0001 | 0.906 |
| **race_ethnic** | 3 | 1 | -0.1927 | 0.0296 | 42.3387 | <.0001 | 0.825 |
| **MCOregion** | 0 | 1 | -0.2247 | 0.0291 | 59.6501 | <.0001 | 0.799 |
| **MCOregion** | 1 | 1 | -0.0463 | 0.0261 | 3.1423 | 0.0763 | 0.955 |
| **MCOregion** | 2 | 1 | -0.0159 | 0.0340 | 0.2190 | 0.6398 | 0.984 |
| **MCOregion** | 3 | 1 | 0.1725 | 0.0331 | 27.1006 | <.0001 | 1.188 |
| **enrl_days** |  | 1 | -0.00317 | 0.000165 | 368.0156 | <.0001 | 0.997 |
| **rurality_residence** | 0 | 1 | -0.1183 | 0.0287 | 17.0071 | <.0001 | 0.888 |
| **rurality_residence** | 1 | 1 | -0.0208 | 0.0237 | 0.7680 | 0.3808 | 0.979 |
| **CCWtotal** |  | 1 | 0.00879 | 0.000242 | 1314.5140 | <.0001 | 1.009 |
| **Children_and_familie** |  | 1 | -1.0030 | 0.0544 | 339.4372 | <.0001 | 0.367 |
| **Disabled** |  | 1 | -0.6746 | 0.0542 | 155.1421 | <.0001 | 0.509 |
| **Chronically_Ill** |  | 1 | -0.5708 | 0.3104 | 3.3811 | 0.0659 | 0.565 |
| **Healthy_Horizons** |  | 1 | -1.7164 | 0.0676 | 643.8628 | <.0001 | 0.180 |
| **PrimaryCareFreq_annu** |  | 1 | -0.2219 | 0.0141 | 247.6821 | <.0001 | 0.801 |

Except for non-Hispanic White (p-value=0.6585), MCO Region of New East (p-value=0.0750), MCO Region of New West (p-value=0.6455), residence of metro counties of <1 million population (p-value=0.3691), and additional basis of eligibility of Chronically Ill (p-value=0.0657), all other covariates are significantly associated with continuous enrollment in Medicaid from 2015 to 2019.

| **Association of Predicted Probabilities and Observed Responses** | | | |
| --- | --- | --- | --- |
| **Percent Concordant** | 74.0 | **Somer’s D^[[2]](#footnote-2)^** | 0.481 |
| **Percent Discordant** | 25.9 | **Gamma** | 0.481 |
| **Percent Tied^[[3]](#footnote-3)^** | 0.0 | **Tau-a^[[4]](#footnote-4)^** | 0.153 |
| **Pairs** | 973,133,371 | **c** | 0.741 |

A pair of observations with different observed responses is said to be concordant if the observation with the lower ordered response value ($CONTENROLL$ = 0) has a lower predicted mean score than the observation with the higher ordered response value ($CONTENROLL$ = 1). Discordant pairs, on the other hand, include the observations with the lower ordered response value with a higher predicted mean score than the observation with the higher ordered response value. In our data, 74% of the pairs are concordant; 26% are discordant with no tied pairs.

Somer’s D is used to determine the strength and direction of relation between pairs of variables. Its values range from -1.0 (all pairs disagree) to 1.0 (all pairs agree). As the value of Somer’s D in our data is 0.481, equaling the difference between the percent concordant and the percent discordant divided by 100. The Goodman-Kruskal Gamma method does not penalize for ties on either variable. Its values range from -1.0 (no association) to 1.0 (perfect association). Kendall’s Tau-a is a modification of Somer’s D that takes into the account the difference between the number of possible paired observations and the number of paired observations with a different response. c is equivalent to the well-known measure ROC. c ranges from 0.5 to 1, where 0.5 corresponds to the model randomly predicting the response, and a 1 corresponds to the model perfectly discriminating the response.

**ROC Curve for Model**

Area under the curve = 0.7407


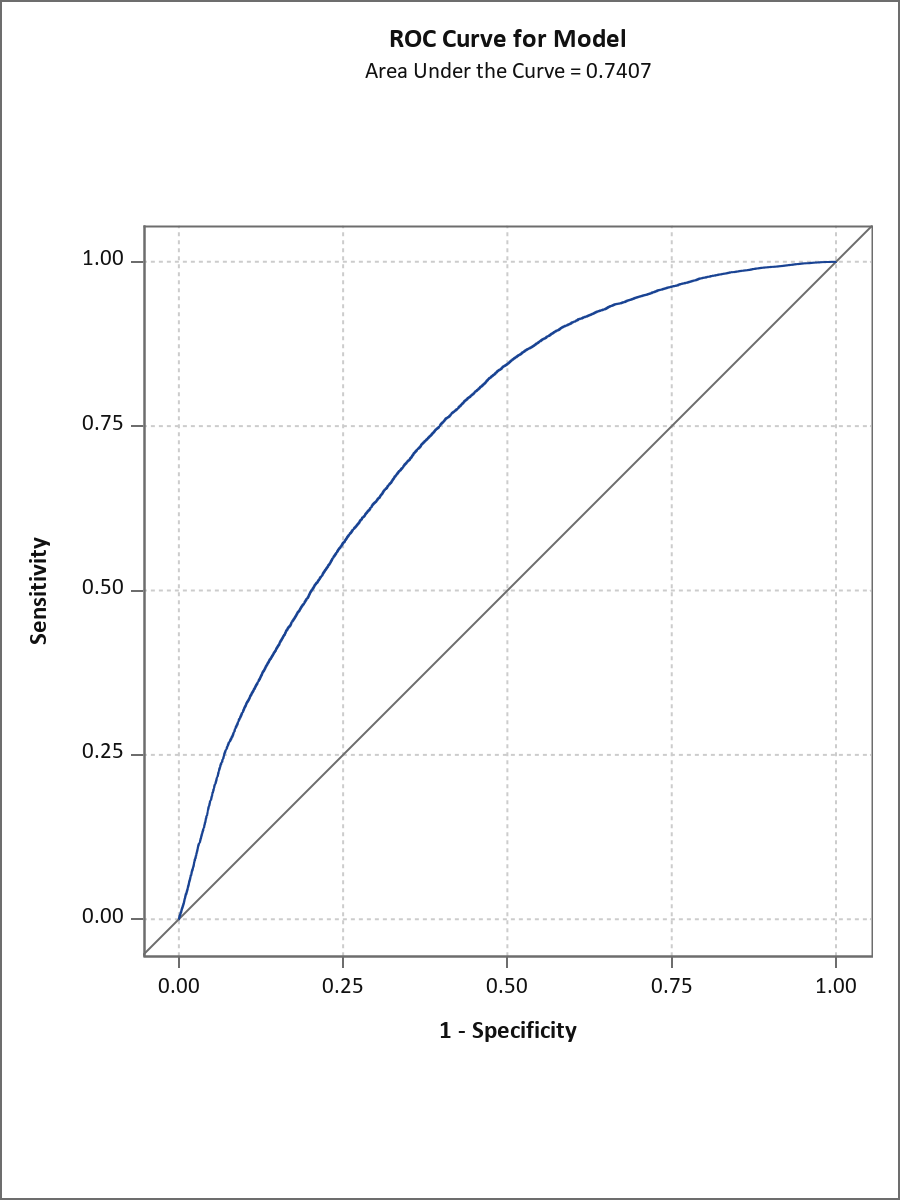


Step 2. Calculate adjusted predicted probabilities (*PredProb*) of staying enrolled in Medicaid from 2015 to 2019 based on Model 1 for each enrollee.

**Histogram of adjusted predicted probabilities of staying enrolled in Medicaid**


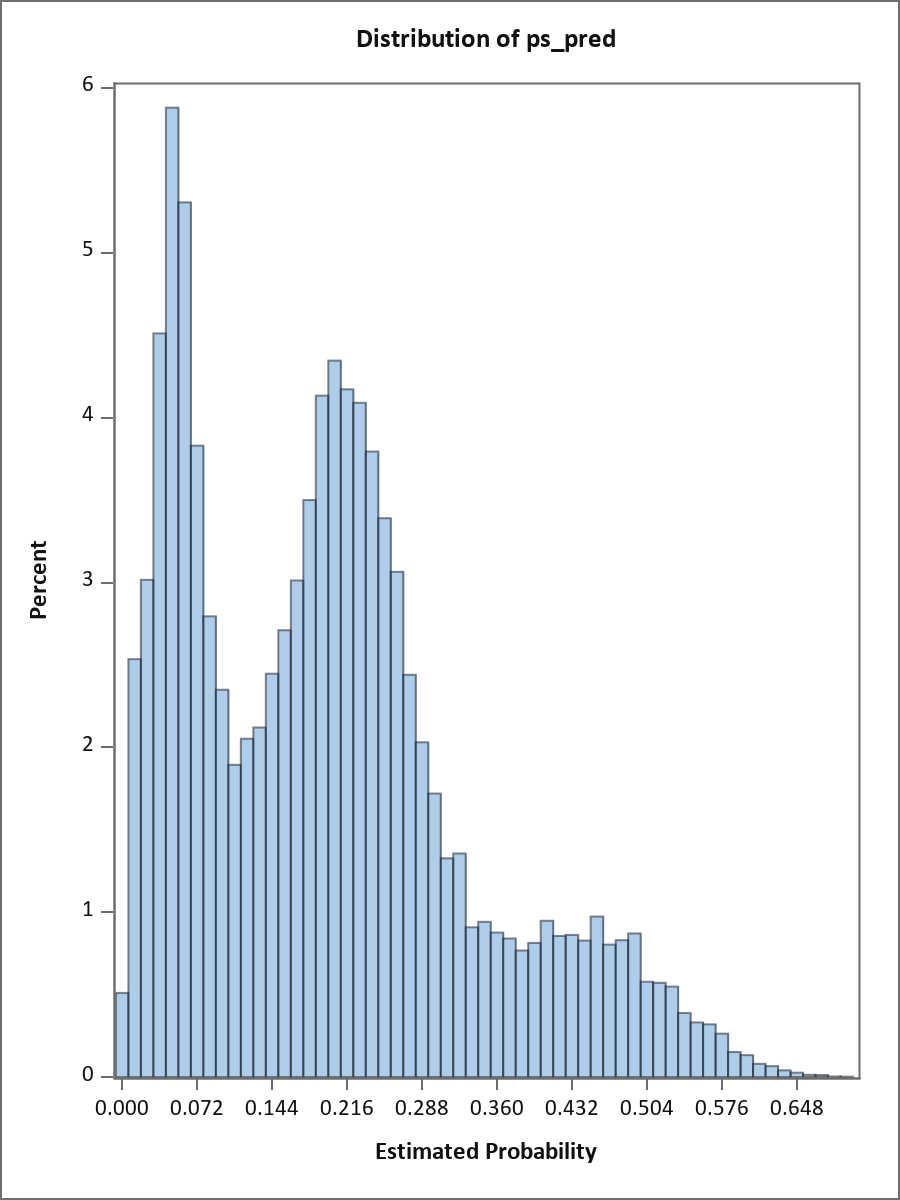


Step 3. Calculate IPCW based on PredProb and $CONTENROLL$.

$$IPCW=\frac{1}{PredProb} if CONTENROLL=1$$

$$IPCW=\frac{1}{1-PredProb}if CONTENROLL=0$$

# Figure S1. Absolute Standardized Differences between Unweighted and Weighted Means for All Covariates for IPCW


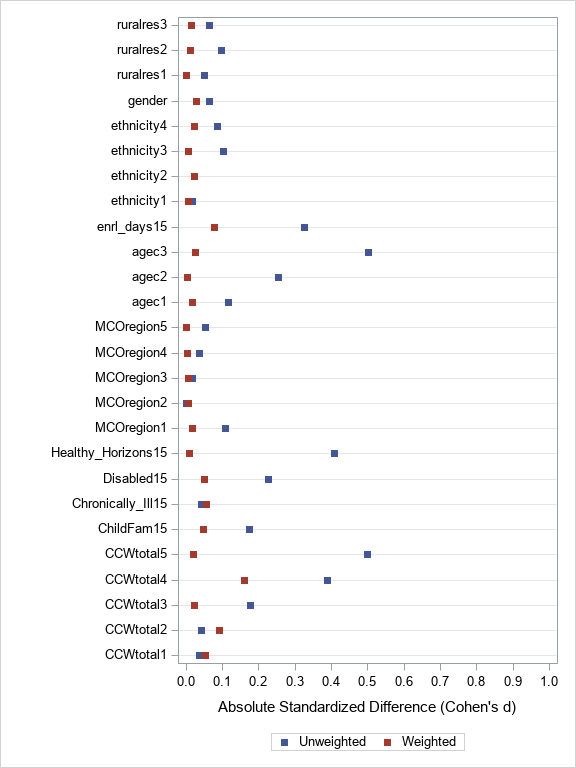


This figure compares differences between the unweighted difference and the weighted difference for each level of all covariates.

# Figure S2. Unweighted, Weighted Proportions, and the Differences of Age among Newly Enrolled Medicaid Enrollees by Continuous Enrollment from 2015 to 2019

**NOTE** Weighted difference<0.10 indicates that the covariate is balanced with inverse probability weighting regarding continuous enrollment from 2015 to 2019. Age is well balanced after weighting across all levels.

# Figure S3. Unweighted, Weighted Proportions, and the Differences of Sex among Newly Enrolled Medicaid Enrollees by Continuous Enrollment from 2015 to 2019

**NOTE** Weighted difference<0.10 indicates that the covariate is balanced with inverse probability weighting regarding continuous enrollment from 2015 to 2019. Sex is well balanced after weighting.

# Figure S4. Unweighted, Weighted Proportions, and the Differences of Race/ethnicity Newly Enrolled Medicaid Enrollees by Continuous Enrollment from 2015 to 2019

**NOTE** Weighted difference<0.10 indicates that the covariate is balanced with inverse probability weighting regarding continuous enrollment from 2015 to 2019. Race/ethnicity is well balanced after weighting across all levels.

# Figure S5. Unweighted, Weighted Proportions, and the Differences of Additional Basis of Eligibility Newly Enrolled Medicaid Enrollees by Continuous Enrollment from 2015 to 2019

**NOTE** Weighted difference<0.10 indicates that the covariate is balanced with inverse probability weighting regarding continuous enrollment from 2015 to 2019. Children and Families, Chronically Ill, Disabled, and Healthy Horizons are well balanced after weighting.

# Figure S6. Unweighted, Weighted Proportions, and the Differences of Managed Care Organization (MCO) Region Newly Enrolled Medicaid Enrollees by Continuous Enrollment from 2015 to 2019

**NOTE** Weighted difference<0.10 indicates that the covariate is balanced with inverse probability weighting regarding continuous enrollment from 2015 to 2019. MCO Region is well balanced after weighting across all levels.

# Figure S7. Unweighted, Weighted Proportions, and the Differences of Total Number of Chronic Conditions at Enrollment Newly Enrolled Medicaid Enrollees by Continuous Enrollment from 2015 to 2019

**NOTE** Weighted difference<0.10 indicates that the covariate is balanced with inverse probability weighting regarding continuous enrollment from 2015 to 2019. Number of chronic conditions at enrollment is not well balanced across the levels after weighting as the weighted difference is 0.161 for those with number of chronic conditions of 5+.

# Figure S8. Unweighted, Weighted Proportions, and the Differences of Mean Number of Enrolled Days in 2015 Newly Enrolled Medicaid Enrollees by Continuous Enrollment from 2015 to 2019

**NOTE** Weighted difference<0.10 indicates that the covariate is balanced with inverse probability weighting regarding continuous enrollment from 2015 to 2019. Total enrollment length is not well balanced after weighting as the weighted difference is 0.114.

# Figure S9. Unweighted, Weighted Proportions, and the Differences of Rurality of Residence at Enrollment Newly Enrolled Medicaid Enrollees by Continuous Enrollment from 2015 to 2019

**NOTE** Weighted difference<0.10 indicates that the covariate is balanced with inverse probability weighting regarding continuous enrollment from 2015 to 2019. Rurality of residence is well balanced after weighting across all levels.

# Calculating Inverse Probability of Using Any Primary Care Services (IPTW) from 2015 to 2019

We compared sociodemographic characteristics of 15,547 enrollees who meet exclusion criteria 1 – 10 (Figure 4.1) by use of any primary care services from 2015 to 2019 in Appendix Table 8. Enrollees who used no primary care services from 2015 to 2019 are significantly younger on average as a larger proportion of them are 50-54 years old and a smaller proportion of them are 60-64 years old than those of enrollees who used any primary care services. A larger proportion of these enrollees are female, but this difference is not statistically significant. We observe that a larger proportion of enrollees who used any primary care services is non-Hispanic White while a significantly smaller proportion of non-Hispanic Black than those among enrollees who used no primary care services. No significant differences in prevalence of additional basis of eligibility criteria are observed between the two groups. A significantly larger proportion of enrollees with no primary care services reside in metro counties of >1 million population. A significantly larger proportion of enrollees who used any primary care services identifies Lehigh Capital or New East as their MCO regions while a significantly larger proportion of enrollees who used no primary care services identifies Southeast or Southwest as their MCO regions. A proportion of enrollees whose total enrollment length per calendar year is between 180 and 340 days is higher among enrollees with no primary care services use while a proportion of enrollees whose total enrollment length per calendar year is between 360.1 and 365.2 days is higher among enrollees who used any primary care services. About 9 out of 10 enrollees who did not use any primary care services have missing chronic condition information, which is significantly higher than those with any primary care services use (12%). About 30% of enrollees who used any primary care services have no chronic conditions.

# Table S8. Baseline Characteristics of Newly expansion Medicaid Enrollees in 2015 by Primary Care Services Use from 2015 to 2019

|  | **No Primary Care Services** | **Any Primary Care Services** |
| --- | --- | --- |
| **N** | 4,559 | 10,988 |
| ***%*** | *29.32* | *70.68* |
| **Outcome Measures** |  |  |
| Any CRC testing*** | 4.39 | 42.58 |
| Any stool-based tests^1^*** | 0.53 | 11.06 |
| Any visual tests^2^*** | 3.50 | 35.80 |
| Any stool-based and visual tests*** | 0.43 | 5.63 |
| Standard Colonoscopy*** | 3.46 | 35.69 |
| **Age**** |  |  |
| 50-54 | 49.42 | 46.61 |
| 55-59 | 43.96 | 45.61 |
| 60-64 | 6.62 | 7.78 |
| **Sex** |  |  |
| Female | 46.24 | 44.77 |
| Male | 53.76 | 55.23 |
| **Race/Ethnicity***** |  |  |
| Non-Hispanic White | 58.57 | 66.86 |
| Non-Hispanic Black | 28.81 | 12.66 |
| Hispanic | 4.76 | 6.93 |
| Other | 8.86 | 13.55 |
| **Additional basis of eligibility** |  |  |
| Children and Families | 2.41 | 2.68 |
| Disabled | 2.79 | 2.66 |
| Chronically Ill | 0.04 | 0.09 |
| Healthy Horizons | 1.49 | 1.57 |
| **Rurality of residence***** |  |  |
| Metro counties of >1 million population | 58.72 | 51.88 |
| Metro counties <1 million population | 27.13 | 35.10 |
| Nonmetro (Urban or completely rural counties) | 14.15 | 13.01 |
| **MCO regions***** |  |  |
| Lehigh Capital | 14.74 | 20.15 |
| New East | 12.66 | 15.52 |
| New West | 7.33 | 7.09 |
| Southeast | 39.04 | 34.49 |
| Southwest | 26.23 | 22.75 |
| **Total enrollment days per calendar year***** |  |  |
| 180 – 340 | 21.10 | 18.95 |
| 340.1 – 350 | 17.11 | 16.02 |
| 350.1 – 360 | 25.20 | 25.07 |
| 360.1 – 365.2 | 36.59 | 39.96 |
| **Total number of chronic conditions***** |  |  |
| 0 | 7.72 | 29.36 |
| 1 – 2 | 2.59 | 29.99 |
| 3 – 4 | 1.23 | 16.77 |
| 5 or more | 0.97 | 12.17 |
| Missing | 87.50 | 11.71 |
| **NOTE** If an enrollee had at least one claim for primary care services from 2015 to 2019, we categorized this enrolle with any primary care services use.  Except for Total Enrollment Days, percentage of each factor is presented.  ^1^This group excludes beneficiaries who had any visual CRC tests.  ^2^This group excludes beneficiaries who had any stool-based CRC tests  ***p<0.001, **p<0.01, *p<0.05 | | |

To adjust for the differences in these observed characteristics by use of primary care services from 2015 to 2019, we implemented the following steps to calculate the inverse probability treatment weight, *IPTW*, for each enrollee in SAS 9.4.

Step 1. Run a multivariable logistic regression model estimating the likelihood of using any primary care services from 2015 to 2019 (Model 2)

$$Y(\Pr\left( {USEDPRIMARYCARESERVICES}_{i}=1 \right))=\beta_{0}+\beta_{1}{Age}_{i}+\beta_{2}{Sex}_{i}+\beta_{3}{RaceEthnicity}_{i}+\beta_{4}{ChildrenandFamilies}_{i}+{\beta_{5}{Disabled}_{i}+\beta_{6}{ChronicallyIll}_{i}+\beta_{7}{HealthyHorizon}_{i}+\beta}_{8}{RuralityResidence}_{i}+\beta_{9}{MCORegion}_{i}+\beta_{10}{TotalEnrollLength}_{i}+\beta_{11}{TotalChronicConditions}_{i}$$

 Model 2

In Model 2, $USEDPRIMARYCARESERVICES$ is a binary measure of whether an enrollee, *i*, uses any primary care services from 2015 to 2019. Except for *TotalEnrollLength,* a continuous variable*,* all other covariates are either binary or categorical.

The following sets of outputs provide the various diagnostics tests of Model 2.

| **Model Fit Statistics** | | |
| --- | --- | --- |
| **Criterion** | **Intercept Only** | **Intercept and Covariates** |
| **AIC** | 18,814.715 | 10,115.576 |
| **SC** | 18,822.366 | 10,260.957 |
| **-2 Log L** | 18,812.715 | 10,077.576 |

Akaike Information Criterion (AIC) and Schwarz Criterion (SC) are used to assess the model fit. AIC compares non-nested models; the model with the smallest AIC is desired. Both AIC and SC penalize the number of predictors in the model. Based on the AIC and SC values above, the model with intercept and covariates is preferred.

| **Testing Global Null Hypothesis: BETA=0** | | | |
| --- | --- | --- | --- |
| **Test** | **Chi-Square** | **DF** | **Pr>ChiSq** |
| **Likelihood Ratio** | 8,735.1386 | 18 | <.0001 |
| **Score** | 8,327.0503 | 18 | <.0001 |
| **Wald** | 5,282.1063 | 18 | <.0001 |

The likelihood ratio, score, and Wald tests evaluate the null hypothesis that at least one of the predictors’ regression coefficient is not equal to zero in the model. As all three tests reject the null hypothesis, at least one of the predictors’ regression coefficient is not equal to zero in Model 2.

| **Analysis of Maximum Likelihood Estimates** | | | | | | | |
| --- | --- | --- | --- | --- | --- | --- | --- |
| **Parameter** |  | **DF** | **Estimate** | **Standard Error** | **Wald Chi-Square** | **Pr>ChiSq** | **Exp(Est)** |
| **Intercept** |  | 1 | 2.0785 | 0.6221 | 11.1630 | 0.0008 | 7.992 |
| **agec** | 0 | 1 | -0.0768 | 0.0433 | 3.1542 | 0.0757 | 0.926 |
| **agec** | 1 | 1 | 0.0447 | 0.0435 | 1.0545 | 0.3045 | 1.046 |
| **gender** |  | 1 | -0.0968 | 0.0523 | 3.4292 | 0.0641 | 0.908 |
| **race_ethnic** | 1 | 1 | 0.2420 | 0.0494 | 24.0110 | <.0001 | 1.274 |
| **race_ethnic** | 2 | 1 | -0.6328 | 0.0588 | 115.7913 | <.0001 | 0.531 |
| **race_ethnic** | 3 | 1 | 0.0625 | 0.0875 | 0.5100 | 0.4751 | 1.064 |
| **MCOregion** | 0 | 1 | -0.00290 | 0.0797 | 0.0013 | 0.9710 | 0.997 |
| **MCOregion** | 1 | 1 | 0.1671 | 0.0717 | 5.4271 | 0.0198 | 1.182 |
| **MCOregion** | 2 | 1 | -0.1858 | 0.0912 | 4.1482 | 0.0417 | 0.830 |
| **MCOregion** | 3 | 1 | 0.1356 | 0.0900 | 2.2722 | 0.1317 | 1.145 |
| **totalenrldays** |  | 1 | 0.000435 | 0.000353 | 1.5163 | 0.2182 | 1.000 |
| **rurality_residence** | 0 | 1 | 0.0141 | 0.0770 | 0.0336 | 0.8546 | 1.014 |
| **rurality_residence** | 1 | 1 | 0.1977 | 0.0633 | 9.7712 | 0.0018 | 1.219 |
| **CCWtotal** |  | 1 | -0.0407 | 0.000565 | 5184.2546 | <.0001 | 0.960 |
| **ChildFam15** |  | 1 | -0.0190 | 0.1631 | 0.0136 | 0.9071 | 0.981 |
| **Disabled15** |  | 1 | -0.3310 | 0.1603 | 4.2630 | 0.0390 | 0.718 |
| **Chronically_Ill15** |  | 1 | -0.8420 | 0.9528 | 0.7810 | 0.3768 | 0.431 |
| **Healthy_Horizons15** |  | 1 | -0.1313 | 0.2132 | 0.3794 | 0.5379 | 0.877 |

Except for non-Hispanic White (p-value=<.0001), Hispanic (p-value=<.0001), residence of metro counties of <1 million population (p-value=0.0198), MCO region of New East (p-value=0.0198), MCO region of New West (p-value=0.0417), residence of nonmetro counties (p-value=0.0018), total number of chronic conditions (p-value<.0001), and additional basis of eligibility of Disabled (p-value=0.0390), all other covariates are not significantly associated with use of any primary care services in Medicaid from 2015 to 2019.

| **Association of Predicted Probabilities and Observed Responses** | | | |
| --- | --- | --- | --- |
| **Percent Concordant** | 89.5 | **Somer’s D^[[5]](#footnote-5)^** | 0.791 |
| **Percent Discordant** | 10.5 | **Gamma** | 0.791 |
| **Percent Tied^[[6]](#footnote-6)^** | 0.0 | **Tau-a^[[7]](#footnote-7)^** | 0.328 |
| **Pairs** | 50,094,292 | **c** | 0.895 |

Concordant pairs indicate pairs of observations in which the observation with the lower ordered response value ($USEDPRIMARYCARESERVICES$ = 0) has a lower predicted mean score than the observation with the higher ordered response value ($USEDPRIMARYCARESERVICES$ = 1). Discordant pairs, on the other hand, include the observations with the lower ordered response value with a higher predicted mean score than the observation with the higher ordered response value. In our data, 90.5% of the pairs are concordant; 10.5% are discordant with no tied pairs. Somer’s D is used to determine the strength and direction of relation between pairs of variables. Its values range from -1.0 (all pairs disagree) to 1.0 (all pairs agree). As the value of Somer’s D in our data is 0.791, equaling the difference between the percent concordant and the percent discordant divided by 100. The Goodman-Kruskal Gamma method does not penalize for ties on either variable. Its values range from -1.0 (no association) to 1.0 (perfect association). Kendall’s Tau-a is a modification of Somer’s D that takes into the account the difference between the number of possible paired observations and the number of paired observations with a different response. c is equivalent to the ROC and ranges from 0.5 to 1, where 0.5 corresponds to the model randomly predicting the response, and a 1 corresponds to the model perfectly discriminating the response.

**ROC Curve for model**

Area under the curve = 0.8905


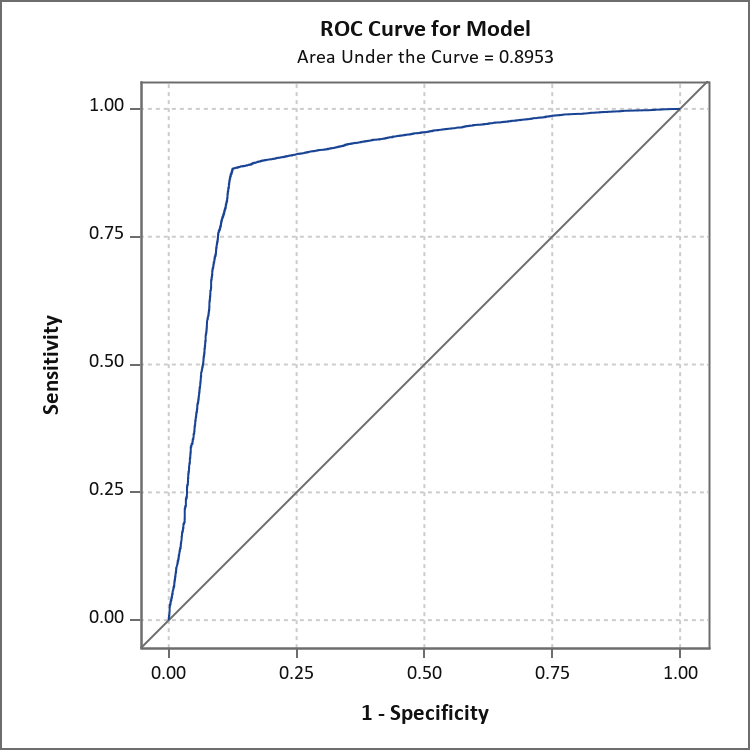


Step 2. Calculate adjusted predicted probabilities (*PredProb*) of using any primary care services from 2015 to 2019 based on Model 2 for each enrollee.

**Histogram of adjusted predicted probabilities of using any primary care services from 2015 to 2019**


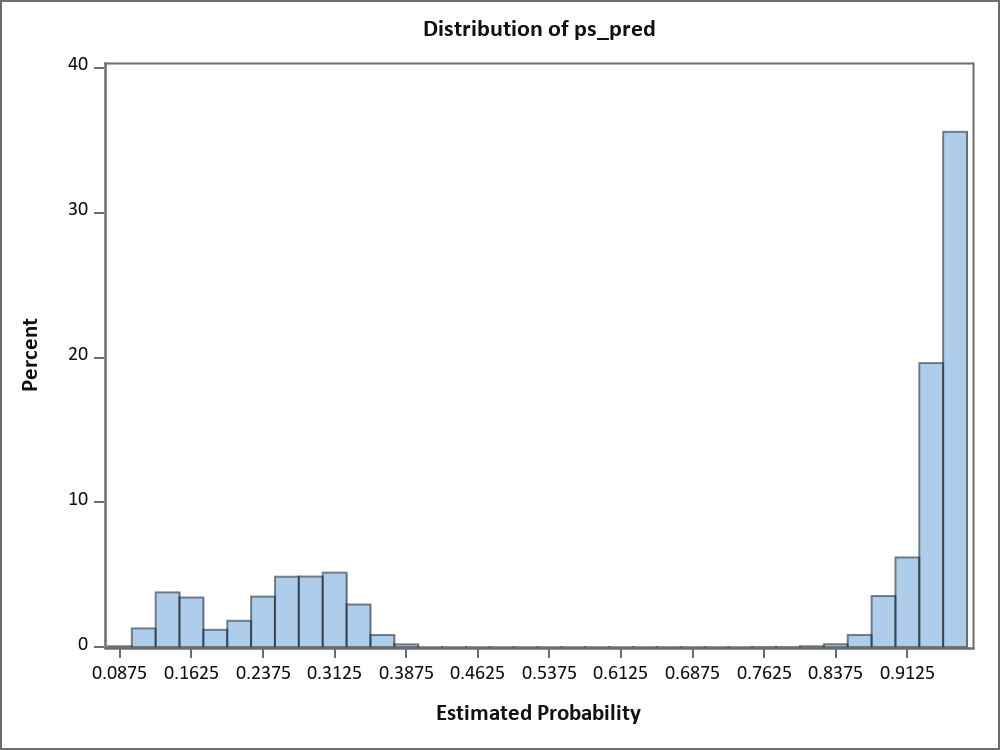


Step 3. Calculate IPTW based on PredProb and $USEDPRIMARYCARESERVICES$

$$IPTW=\frac{1}{PredProb} if USEDPRIMARYCARESERVICES=1$$

$$IPTW=\frac{1}{1-PredProb}if USEDPRIMARYCARESERVICES=0$$

# Figure S10. Absolute Standardized Differences between Unweighted and Weighted Means for All Covariates for IPTW

*
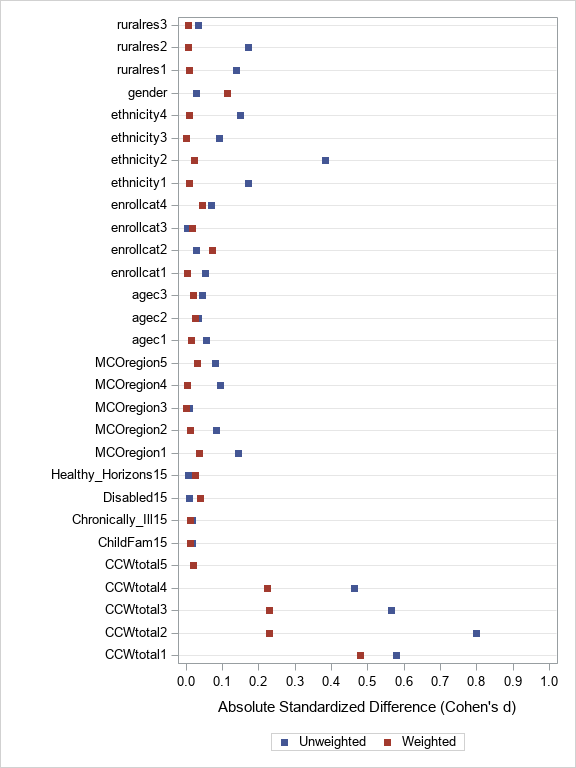
*

**NOTE** This figure compares differences between the unweighted difference and the weighted difference for each level of all covariates.

# Figure S11. Unweighted, Weighted Proportions, and the Differences of Age among Newly Continuously Enrolled Medicaid Enrollees by Primary Care Services Use from 2015 to 2019

**NOTE** Weighted difference<0.10 indicates that the covariate is balanced with inverse probability weighting regarding using any primary care services from 2015 to 2019. Age is well balanced after weighting across all levels.

# Figure S12. Unweighted, Weighted Proportions, and the Differences of Sex among Newly Continuously Enrolled Medicaid Enrollees by Primary Care Services Use from 2015 to 2019

**NOTE** Weighted difference<0.10 indicates that the covariate is balanced with inverse probability weighting regarding using any primary care services from 2015 to 2019. Sex is not well balanced after weighting as the weighted difference is 0.115.

# Figure S13. Unweighted, Weighted Proportions, and the Differences of Race/Ethnicity among Newly Continuously Enrolled Medicaid Enrollees by Primary Care Services Use from 2015 to 2019

**NOTE** Weighted difference<0.10 indicates that the covariate is balanced with inverse probability weighting regarding using any primary care services from 2015 to 2019. Race/ethnicity is well balanced after weighting across all levels.

# Figure S14. Unweighted, Weighted Proportions, and the Differences of Additional Basis of Eligibility among Newly Continuously Enrolled Medicaid Enrollees by Primary Care Services Use from 2015 to 2019

**NOTE** Weighted difference<0.10 indicates that the covariate is balanced with inverse probability weighting regarding using any primary care services from 2015 to 2019. Children and Families, Chronically Ill, Disabled, and Healthy Horizons are well balanced after weighting.

# Figure S15. Unweighted, Weighted Proportions, and the Differences of Managed Care Organization (MCO) Region among Newly Continuously Enrolled Medicaid Enrollees by Primary Care Services Use from 2015 to 2019

**NOTE** Weighted difference<0.10 indicates that the covariate is balanced with inverse probability weighting regarding using any primary care services from 2015 to 2019. MCO Region is well balanced after weighting across all levels.

# Figure S16. Unweighted, Weighted Proportions, and the Differences of Total Number of Chronic Conditions at Enrollment among Newly Continuously Enrolled Medicaid Enrollees by Primary Care Services Use from 2015 to 2019

**NOTE** Weighted difference<0.10 indicates that the covariate is balanced with inverse probability weighting regarding using any primary care services from 2015 to 2019. Number of chronic conditions at enrollment is not well balanced after weighting as only the weighted difference among the enrollees with missing information for chronic conditions is <0.10.

# Figure S17. Unweighted, Weighted Proportions, and the Differences of Total Enrollment Length per Calendar Year (Days) among Newly Continuously Enrolled Medicaid Enrollees by Primary Care Services Use from 2015 to 2019

**NOTE** Weighted difference<0.10 indicates that the covariate is balanced with inverse probability weighting regarding using any primary care services from 2015 to 2019. Total enrollment length per calendar year is well balanced after weighting across all levels.

# Figure S18. Unweighted, Weighted Proportions, and the Differences of Rurality of Residence at Enrollment among Newly Continuously Enrolled Medicaid Enrollees by Primary Care Services Use from 2015 to 2019

**NOTE** Weighted difference<0.10 indicates that the covariate is balanced with inverse probability weighting regarding using any primary care services from 2015 to 2019. Rurality of residence is well balanced after weighting across all levels.

# Figure S19. Distribution of the Composite Inverse Probability Study Weights (IPSW) based on Inverse Probability Censoring Weights (IPCWs) and Inverse Probability Treatment Weights (IPTWs)


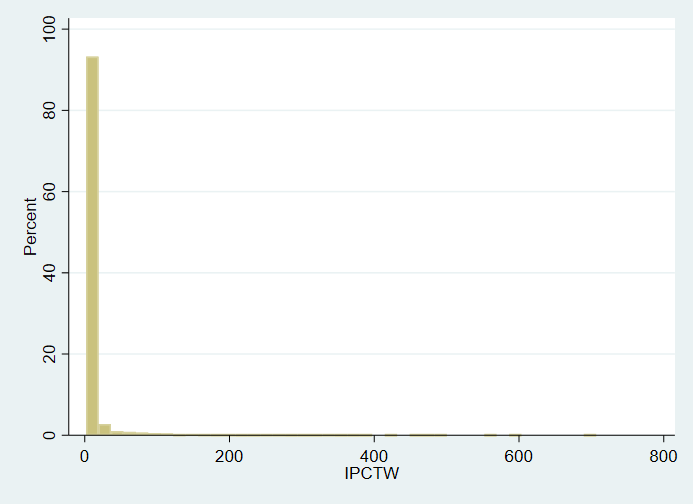


**NOTE IPSW** stands for the product of IPTWs and IPCWs for the analytic sample. Mean of IPCTW is 9.38 while median is 4.47. The range of IPCTWs is from 1.91 to 706.64, with a variance of 543.11 and a kurtosis of 195.67.

# Table S9. Unweighted Individual- and County-level Characteristics of Newly Enrolled Expansion Population among Medicaid Enrollees in Pennsylvania

|  | **(1)** | **(2)** | **(3)** | **(4)** | **(5)** | **(6)** |
| --- | --- | --- | --- | --- | --- | --- |
|  | **Overall** | **No CRC tests** | **Any CRC tests** | **Stool-based Tests Alone** | **Visual Tests Alone** | **Standard Colonoscopy** |
| **Unweighted N** | 15,439 | 10,604 | 4,835 | 800 | 3,643 | 3,624 |
| *Unweighted %* |  | *68.68* | *31.32* | *7.02* | *25.57* | *25.47* |
| ***Individual-level Characteristics*** | | | | | | |
| **Age** |  |  |  |  |  |  |
| 50-54 | 47.43 | 47.69 | 46.91 | 44.13 | 47.54 | 47.57 |
| 55-59 | 45.13 | 44.84 | 45.75 | 48.88 | 44.94 | 44.90 |
| 60-64 | 7.44 | 7.47 | 7.34 | 7.00 | 7.52 | 7.53 |
| **Sex** |  |  | ** | *** |  |  |
| Female | 45.20 | 46.03 | 43.33 | 38.75 | 44.69 | 44.70 |
| Male | 54.80 | 53.97 | 56.67 | 61.25 | 55.31 | 55.30 |
| **Race/Ethnicity** |  |  | *** | *** | *** | *** |
| Non-Hispanic White | 64.43 | 64.96 | 63.16 | 67.50 | 62.12 | 62.09 |
| Non-Hispanic Black | 17.10 | 19.11 | 12.76 | 9.88 | 13.81 | 13.74 |
| Hispanic | 6.29 | 5.26 | 8.48 | 6.50 | 9.14 | 9.16 |
| Other | 12.18 | 10.68 | 15.59 | 16.13 | 14.93 | 15.01 |
| **Rurality of Residence** |  |  | *** | ** | *** | *** |
| Metro counties >1 million population | 53.89 | 54.83 | 51.93 | 61.63 | 49.16 | 49.17 |
| Metro counties <1 million population | 32.77 | 31.67 | 34.99 | 26.13 | 37.83 | 37.83 |
| Nonmetro counties | 13.35 | 13.50 | 13.07 | 12.25 | 13.01 | 13.00 |
| **MCO Regions** |  |  | *** | *** | *** | *** |
| Lehigh Capital | 18.56 | 17.46 | 20.87 | 15.63 | 22.70 | 22.71 |
| New East | 14.68 | 14.51 | 14.97 | 11.75 | 15.70 | 15.70 |
| New West | 7.16 | 7.20 | 7.05 | 4.63 | 7.74 | 7.73 |
| Southeast | 35.83 | 36.07 | 35.33 | 43.88 | 33.49 | 33.47 |
| Southwest | 23.77 | 24.75 | 21.78 | 24.13 | 20.37 | 20.39 |
| **Additional Eligibility Criteria** |  |  |  |  |  |  |
| Children and Families | 2.61 | 2.60 | 2.56 | 2.63 | 2.69 | 2.62 |
| Disabled | 2.70 | 2.71 | 2.59 | 2.75 | 2.64 | 2.59 |
| Chronically Ill | 0.08 | 0.08 | 0.08 | 0.00 | 0.08 | 0.08 |
| Healthy Horizons | 1.54 | 1.58 | 1.47 | 1.50 | 1.54 | 1.55 |
| **Total Enrollment Days per Calendar Year** |  |  | *** |  | *** | *** |
| 180 – <340 | 19.58 | 20.45 | 17.66 | 19.50 | 17.46 | 17.52 |
| 340 – <350 | 16.34 | 16.79 | 15.43 | 14.12 | 15.29 | 15.26 |
| 350 – <360 | 25.11 | 25.38 | 24.47 | 25.50 | 24.57 | 24.61 |
| 360 – <365 | 38.97 | 37.38 | 42.44 | 40.88 | 42.68 | 42.60 |
| **Total Number of Chronic Conditions** |  |  | *** | *** | *** | *** |
| 0 | 23.01 | 21.06 | 27.40 | 31.37 | 26.65 | 26.71 |
| 1 – 2 | 21.95 | 17.40 | 31.75 | 28.88 | 32.53 | 32.51 |
| 3 – 4 | 12.21 | 9.05 | 19.03 | 17.63 | 19.16 | 19.18 |
| >5 | 8.88 | 6.49 | 13.71 | 11.75 | 14.03 | 13.96 |
| Missing | 33.94 | 46.00 | 8.11 | 10.38 | 7.63 | 7.64 |
| **Frequency of Primary Care Services Use per Calendar** **Year** |  |  | *** | *** | *** | *** |
| 0 | 29.32 | 41.06 | 4.14 | 2.88 | 4.34 | 4.30 |
| 1 – 2 | 38.39 | 26.22 | 64.16 | 64.63 | 62.86 | 62.97 |
| 2 – 4 | 16.15 | 15.18 | 18.43 | 19.38 | 18.67 | 18.63 |
| >4 | 16.14 | 17.54 | 13.28 | 13.13 | 14.14 | 14.10 |
| ***County-level Characteristics*** | | | | | | |
|  |  |  | * | *** |  |  |
| **Median Household Income** | 53,603.54  (53,404.79, 53,802.30) | 53,436.23 (53,195.16, 53,677.30) | 53,910.83 (53,556.10, 54,265.56) | 55,474.35 (54,502.46, 56,446.24) | 53,500.89 (53,106.70, 53,895.08) | 53,488.40 (53,093.60, 53,883.20) |
| **Percent living under poverty** |  |  | * |  |  |  |
|  | 14.93  (14.83, 15.02) | 15.01  (14.89, 15.13) | 14.77  (14.60, 14.95) | 14.58  (14.13, 15.03) | 14.84  (14.64, 15.04) | 14.85  (14.65, 15.05) |
| **Percent living in urban areas** |  |  |  | *** |  |  |
|  | 79.32  (78.95, 79.68) | 79.24  (78.80, 79.68) | 79.48  (78.84, 80.12) | 82.50  (80.97, 84.04) | 78.80  (78.06, 79.53) | 75.42  (69.10, 81.73) |
| **Percent Unemployed, 16+** |  |  | * | * | * | * |
|  | 5.52  (5.50, 5.53) | 5.54  (5.52, 5.56) | 5.48  (5.45, 5.51) | 5.46  (5.38, 5.53) | 5.49  (5.45, 5.52) | 5.49  (5.46, 5.52) |
| **Percent <High School Diploma, 25+** |  |  |  | * | * | * |
|  | 11.75  (11.69, 11.82) | 11.74  (11.66, 11.82) | 11.79  (11.67, 11.90) | 11.36  (11.06, 11.66) | 11.93  (11.80, 12.07) | 11.93  (11.80, 12.07) |
| **Percent Non-Hispanic White** | 73.41  (73.07, 73.75) | 73.37  (72.96, 73.78) | 73.45  (72.85, 74.04) | 71.87  (70.41, 73.34) | 73.68  (72.99, 74.37) | 73.67  (72.98, 74.36) |
| **# of PCPs per 1000 persons** |  |  |  | *** |  |  |
|  | 0.78  (0.77, 0.78) | 0.78  (0.77, 0.78) | 0.78  (0.77, 0.79) | 0.82  (0.80, 0.83) | 0.77  (0.76, 0.78) | 0.77  (0.76, 0.78) |
| **# of Specialists per 1000 persons** |  |  |  | * |  |  |
|  | 2.51  (2.48, 2.54) | 2.51  (2.48, 2.54) | 2.51  (2.45, 2.56) | 2.67  (2.56, 2.77) | 2.47  (2.40, 2.54) | 2.47  (2.40, 2.54) |
| **# of Short-term General Hospital Beds per 1000 persons** | 2.93  (2.91, 2.96) | 2.94  (2.91, 2.97) | 2.92  (2.87, 2.97) | 2.90  (2.82, 2.98) | 2.93  (2.87, 2.99) | 2.93  (2.87, 2.99) |
| **# of FQHCs per 1000 persons** | 0.02  (0.02, 0.02) | 0.02  (0.02, 0.02) | 0.02  (0.02, 0.02) | 0.02  (0.02, 0.02) | 0.02  (0.02, 0.02) | 0.03  (0.02, 0.04) |
| **# of RHCs per 1000 persons** |  |  |  | ** |  |  |
|  | 0.01  (0.01, 0.01) | 0.01  (0.01, 0.01) | 0.01  (0.00, 0.01) | 0.01  (0.00, 0.01) | 0.01  (0.01, 0.01) | 0.01  (0.00, 0.01) |
| **CRC**=Colorectal Cancer; **MCO**=Managed Care Organizations; **PCP**=Primary Care Physicians; **FQHC**=Federally Qualified Health Centers; **RHC**=Rural Health Centers  **NOTE** Percentage of each individual-level factor is presented. Estimates for county-level factors include 95% confidence interval.  Any CRC tests include any stool-based tests (gFOBT, FIT, or FIT-DNA) or any visual tests (standard colonoscopy, flexible sigmoidoscopy, or CT colonography). Enrollees who received any CRC tests include 393 enrollees who received both stool-based tests and visual tests during the study period.  Distributions of each factor in Columns (3) – (6) are compared to distributions of each factor in Column (2).  Regarding rurality of residence, we defined metro counties of <1 million population as counties in metro areas of 250,000 to 1 million population or counties in metro areas of fewer than 250,000 population. We defined non-metro counties as counties with urban population of 20,000 or more, adjacent to a metro area; urban population of 2,500 to 19,999, adjacent to a metro area; urban population of 2,500 to 19,990, not adjacent to a metro area; completely rural or less than 2,500 urban population, adjacent to a metro area; or, completely rural or less than 2,500 urban population, not adjacent to a metro area; and, urban areas with populations ranging from 2,500 to 49,999 that are not part of larger labor market areas.  Regarding frequency of primary care services, we translated multiple claims of primary care services on the same day into a single claim by prioritizing FQHCs/RHCs over other sites of care if they visited both FQHCs/RHCs and physician’s office, outpatient hospitals, independent clinics, or public health clinics; we prioritized non-physician’s office for the enrollees with multiple claims of primary care services on the same day if they visited both a physician’s office and outpatient hospitals, independent clinics, or public health clinics. We counted the number of claims for primary care services prior to the first claim for CRC testing for the enrollees who received any CRC testing as this study examines the association between frequency of primary care services use and CRC testing.  ***p<0.001, **p<0.01, *p<0.05 | | | | | | |

# Table S10. Weighted Individual- and County-level Characteristics of Newly Enrolled Expansion Population among Medicaid Enrollees in Pennsylvania

|  | **(1)** | **(2)** | **(3)** | **(4)** | **(5)** | **(6)** |
| --- | --- | --- | --- | --- | --- | --- |
|  | **Overall** | **No CRC tests** | **Any CRC tests** | **Stool-based Tests Alone** | **Visual Tests Alone** | **Standard Colonoscopy** |
| **Weighted %** | - | 67.95 | 32.05 | 6.16 | 26.85 | 26.69 |
| ***Individual-level Characteristics*** | | | | | | |
| **Age** |  |  |  |  |  |  |
| 50-54 | 42.61 | 42.15 | 43.35 | 42.38 | 44.31 | 44.36 |
| 55-59 | 36.10 | 35.18 | 38.17 | 40.97 | 37.67 | 37.52 |
| 60-64 | 21.29 | 22.66 | 18.47 | 16.65 | 18.20 | 18.12 |
| **Sex** |  |  | *** | * | ** | ** |
| Female | 52.66 | 55.02 | 47.54 | 46.85 | 48.70 | 48.64 |
| Male | 47.34 | 44.98 | 52.46 | 53.15 | 51.30 | 51.36 |
| **Race/Ethnicity** |  |  | *** | * | ** | ** |
| Non-Hispanic White | 65.05 | 66.81 | 61.34 | 60.44 | 61.44 | 61.48 |
| Non-Hispanic Black | 15.47 | 15.89 | 14.59 | 13.83 | 14.94 | 14.85 |
| Hispanic | 8.18 | 6.94 | 10.54 | 8.10 | 11.14 | 11.19 |
| Other | 11.30 | 10.36 | 13.52 | 17.63 | 12.48 | 12.58 |
| **Rurality of residence** |  |  |  | ** | ** | ** |
| Metro counties >1 million population | 51.34 | 52.67 | 48.85 | 65.89 | 45.99 | 45.74 |
| Metro counties <1 million population | 37.09 | 35.71 | 39.90 | 24.50 | 42.18 | 42.36 |
| Nonmetro counties | 11.57 | 11.62 | 11.25 | 9.61 | 11.83 | 11.89 |
| **MCO regions** |  |  |  | ** | ** | ** |
| Lehigh Capital | 23.98 | 22.89 | 26.26 | 15.61 | 27.77 | 27.90 |
| New East | 13.94 | 13.96 | 13.93 | 8.57 | 14.92 | 14.99 |
| New West | 6.72 | 6.14 | 7.52 | 5.25 | 8.14 | 8.17 |
| Southeast | 34.19 | 35.07 | 32.56 | 48.82 | 29.96 | 29.78 |
| Southwest | 21.17 | 21.94 | 19.73 | 21.76 | 19.21 | 19.16 |
| **Additional eligibility criteria** |  |  |  |  |  |  |
| Children and Families | 5.60 | 5.48 | 5.40 | 3.46 | 6.05 | 5.89 |
| Disabled | 6.14 | 5.92 | 6.45 | 7.27 | 6.70 | 6.71 |
| Chronically Ill | 0.39 | 0.48 | 0.21 | 0.00 | 0.26 | 0.26 |
| Healthy Horizons | 6.66 | 6.48 | 7.17 | 9.37 | 7.08 | 7.14 |
| **Total enrollment days per calendar year** |  |  | *** |  | ** | ** |
| 180 – <340 | 16.99 | 17.35 | 16.20 | 19.75 | 15.79 | 15.91 |
| 340 – <350 | 17.21 | 18.66 | 14.09 | 12.78 | 14.20 | 14.11 |
| 350 – <360 | 25.92 | 26.81 | 24.14 | 23.63 | 25.02 | 25.19 |
| 360 – <365 | 39.89 | 37.18 | 45.57 | 43.84 | 44.98 | 44.79 |
| **Total number of chronic conditions** |  |  | *** | *** | *** | *** |
| 0 | 36.50 | 40.22 | 28.84 | 29.38 | 27.44 | 27.60 |
| 1 – 2 | 21.31 | 17.96 | 28.54 | 26.88 | 29.26 | 29.06 |
| 3 – 4 | 12.69 | 9.66 | 19.02 | 18.68 | 19.75 | 19.85 |
| >5 | 10.63 | 8.14 | 15.35 | 11.70 | 16.07 | 15.97 |
| Missing | 18.87 | 24.02 | 8.25 | 13.36 | 7.47 | 7.52 |
| **Frequency of primary care services use per calendar year** |  |  | *** | *** | *** | *** |
| 0 | 46.91 | 54.28 | 31.58 | 20.78 | 32.54 | 32.34 |
| 1 – 2 | 27.46 | 19.54 | 43.69 | 50.63 | 42.22 | 42.39 |
| 2 – 4 | 11.85 | 10.97 | 13.77 | 15.87 | 13.98 | 13.99 |
| >4 | 13.78 | 15.20 | 10.96 | 12.72 | 11.27 | 11.28 |
| ***County-level Characteristics*** | | | | | | |
| **Median household income** |  |  |  |  | * | * |
|  | 54,315.03  (53,799.32, 54,830.74) | 54,593.02  (53,895.13, 55,290.91) | 53,739.91 (53,100.04, 54,379.77) | 54,760.19 (53,124.59, 56,395.79) | 53,463.13  (52,734.03, 54,192.23) | 53,412.20  (52,682.93, 54,141.46) |
| **Percent living in urban areas** |  |  |  | *** |  |  |
|  | 79.38  (78.49, 80.28) | 79.39  (78.26, 80.52) | 79.49  (78.01, 80.96) | 85.51  (83.24, 87.78) | 78.49  (76.91, 80.06) | 78.42  (76.84, 80.01) |
| **Percent Non-Hispanic White** |  |  |  | * |  |  |
|  | 73.67  (72.91, 74.42) | 73.78  (72.86, 74.70) | 73.29  (71.96, 74.61) | 68.53  (65.38, 71.68) | 74.03  (72.54, 75.51) | 74.01  (72.51, 75.51) |
| **Percent unemployed among population of 16+ years** |  |  |  |  |  |  |
|  | 5.43  (5.39, 5.48) | 5.44  (5.39, 5.49) | 5.42  (5.35, 5.49) | 5.51  (5.37, 5.66) | 5.42  (5.35, 5.49) | 5.42  (5.35, 5.50) |
| **Percent in living under poverty** |  |  |  |  | * | * |
|  | 14.51  (14.28, 14.74) | 14.45  (14.15, 14.74) | 14.66  (14.27, 15.05) | 15.29  (14.30, 16.28) | 14.62  (14.19, 15.05) | 14.64  (14.21, 15.07) |
| **Percent <high school diploma among population of 25+ years** |  |  |  |  |  |  |
|  | 11.75  (11.58, 11.91) | 11.67  (11.46, 11.89) | 11.92  (11.67, 12.17) | 11.71  (11.95, 12.37) | 11.99  (11.70, 12.27) | 12.01  (11.72, 12.29) |
| **# of PCPs per 1000 persons** |  |  |  | * |  |  |
|  | 0.78  (0.77, 0.80) | 0.78  (0.77, 0.80) | 0.79  (0.77, 0.81) | 0.82  (0.79, 0.85) | 0.78  (0.76, 0.81) | 0.78  (0.76, 0.81) |
| **# of specialists per 1000 persons** |  |  |  | *** |  |  |
|  | 2.47  (2.39, 2.54) | 2.43  (2.35, 2.51) | 2.57  (2.39, 2.73) | 2.85  (2.68, 3.02) | 2.53  (2.33, 2.74) | 2.54  (2.33, 2.74) |
| **# of short-term general hospital beds per 1000 persons** |  |  |  | ** |  |  |
|  | 2.87  (2.80, 2.93) | 2.82  (2.76, 2.89) | 2.96  (2.82, 3.10) | 3.02  (2.90, 3.15) | 2.97  (2.80, 3.15) | 2.98  (2.81, 3.15) |
| **# of FQHCs per 1000 persons** |  |  |  |  |  |  |
|  | 0.02 | 0.02 | 0.02 | 0.02 | 0.02 | 0.02 |
|  | (0.02, 0.02) | (0.02, 0.02) | (0.02, 0.02) | (0.02, 0.02) | (0.02, 0.02) | (0.02, 0.02) |
| **# of RHCs per 1000 persons** |  |  |  | * |  |  |
|  | 0.01 | 0.01 | 0.01 | 0.01 | 0.01 | 0.01 |
|  | (0.01, 0.01) | (0.01, 0.01) | (0.00, 0.01) | (0.00, 0.01) | (0.01, 0.01) | (0.00, 0.01) |
| **CRC**=Colorectal Cancer; **MCO**=Managed Care Organizations; **PCP**=Primary Care Physicians; **FQHC**=Federally Qualified Health Centers; **RHC**=Rural Health Centers  **NOTE** Percentage of each individual-level factor is presented. Estimates for county-level factors include 95% confidence interval.  All estimates were weighted by a composite inverse probability weighting score based on an enrollee’s propensity to enroll longer in Medicaid and to use primary care services during the study period.  Any CRC tests include any stool-based tests (gFOBT, FIT, or FIT-DNA) or any visual tests (standard colonoscopy, flexible sigmoidoscopy, or CT colonography). Enrollees who received any CRC tests include 393 enrollees who received both stool-based tests and visual tests during the study period.  Distributions of each factor in Columns (3) – (6) are compared to distributions of each factor in Column (2).  Regarding rurality of residence, we defined metro counties of <1 million population as counties in metro areas of 250,000 to 1 million population or counties in metro areas of fewer than 250,000 population. We defined non-metro counties as counties with urban population of 20,000 or more, adjacent to a metro area; urban population of 2,500 to 19,999, adjacent to a metro area; urban population of 2,500 to 19,990, not adjacent to a metro area; completely rural or less than 2,500 urban population, adjacent to a metro area; or, completely rural or less than 2,500 urban population, not adjacent to a metro area; and, urban areas with populations ranging from 2,500 to 49,999 that are not part of larger labor market areas.  Regarding frequency of primary care services, we translated multiple claims of primary care services on the same day into a single claim by prioritizing FQHCs/RHCs over other sites of care if they visited both FQHCs/RHCs and physician’s office, outpatient hospitals, independent clinics, or public health clinics; we prioritized non-physician’s office for the enrollees with multiple claims of primary care services on the same day if they visited both a physician’s office and outpatient hospitals, independent clinics, or public health clinics. We counted the number of claims for primary care services prior to the first claim for CRC testing for the enrollees who received any CRC testing as this study examines the association between frequency of primary care services use and CRC testing.  ***p<0.001, **p<0.01, *p<0.05 | | | | | | |

# Table S11. Unweighted Association (Odds Ratio [95% CI]) between Individual- and County-level Characteristics of Medicaid Enrollees and Receipt of CRC Tests

|  | **Any CRC tests** | | **Stool-based Tests Alone** | | **Visual tests Alone** | | **Standard Colonoscopy** | |
| --- | --- | --- | --- | --- | --- | --- | --- | --- |
|  | **(1)** | **(2)** | **(1)** | **(2)** | **(1)** | **(2)** | **(1)** | **(2)** |
| **Unweighted N** | 15,439 | 15,439 | 11,395 | 11,395 | 14,247 | 14,247 | 14,228 | 14,228 |
| ***Individual-level Characteristics*** | | | | | | | | |
| **Age** |  |  |  |  |  |  |  |  |
| 50-54 | REF | REF | REF | REF | REF | REF | REF | REF |
| 55-59 | 1.02 | 1.02 | 1.16* | 1.15* | 0.99 | 0.99 | 0.98 | 0.98 |
|  | [0.94-1.10] | [0.94-1.10] | [0.99-1.36] | [0.99-1.35] | [0.90-1.08] | [0.90-1.08] | [0.90-1.08] | [0.90-1.08] |
| 60-64 | 0.87* | 0.87* | 0.89 | 0.9 | 0.88 | 0.88 | 0.88 | 0.88 |
|  | [0.74-1.01] | [0.74-1.01] | [0.66-1.21] | [0.66-1.22] | [0.75-1.04] | [0.74-1.04] | [0.74-1.04] | [0.74-1.04] |
| **Male** | 1.20*** | 1.20*** | 1.50*** | 1.51*** | 1.13*** | 1.13*** | 1.13*** | 1.13*** |
|  | [1.11-1.30] | [1.11-1.30] | [1.28-1.75] | [1.29-1.77] | [1.04-1.23] | [1.04-1.24] | [1.04-1.23] | [1.04-1.24] |
| **Race/Ethnicity** |  |  |  |  |  |  |  |  |
| Non-Hispanic White | REF | REF | REF | REF | REF | REF | REF | REF |
| Non-Hispanic Black | 0.98 | 0.95 | 0.54*** | 0.51*** | 1.15** | 1.11 | 1.15* | 1.10 |
|  | [0.86-1.12] | [0.83-1.09] | [0.41-0.71] | [0.38-0.68] | [1.00-1.32] | [0.96-1.29] | [1.00-1.32] | [0.95-1.28] |
| Hispanic | 1.52*** | 1.49*** | 1.07 | 1.01 | 1.65*** | 1.62*** | 1.65*** | 1.62*** |
|  | [1.30-1.79] | [1.26-1.75] | [0.77-1.47] | [0.73-1.41] | [1.39-1.95] | [1.36-1.93] | [1.39-1.96] | [1.36-1.93] |
| Other | 1.42*** | 1.39*** | 1.1 | 1.05 | 1.45*** | 1.43*** | 1.46*** | 1.44*** |
|  | [1.25-1.61] | [1.22-1.58] | [0.88-1.39] | [0.84-1.33] | [1.27-1.67] | [1.25-1.65] | [1.28-1.68] | [1.25-1.66] |
| **Rurality of Residence** |  |  |  |  |  |  |  |  |
| Metro counties  >1 million population | REF | REF | REF | REF | REF | REF | REF | REF |
| Metro counties  <1 million population | 1.05 | 0.90 | 1.05 | 1.12 | 1.09 | 0.87 | 1.09 | 0.87 |
|  | [0.86-1.28] | [0.71-1.15] | [0.72-1.54] | [0.68-1.82] | [0.87-1.35] | [0.67-1.14] | [0.87-1.35] | [0.66-1.13] |
| Nonmetro counties | 1.05 | 0.95 | 1.21 | 2.19*** | 1.03 | 0.78* | 1.03 | 0.78* |
|  | [0.87-1.27] | [0.73-1.23] | [0.85-1.73] | [1.30-3.67] | [0.83-1.27] | [0.59-1.03] | [0.83-1.27] | [0.58-1.03] |
| **MCO Regions** |  |  |  |  |  |  |  |  |
| Lehigh Capital | REF | REF | REF | REF | REF | REF | REF | REF |
| New East | 1.01 | 1.02 | 0.95 | 0.96 | 1.01 | 0.96 | 1.01 | 0.96 |
|  | [0.87-1.17] | [0.81-1.28] | [0.70-1.30] | [0.61-1.53] | [0.87-1.18] | [0.75-1.24] | [0.87-1.18] | [0.74-1.23] |
| New West | 0.87 | 0.81 | 0.66* | 0.68 | 0.92 | 0.81 | 0.92 | 0.79 |
|  | [0.72-1.05] | [0.59-1.12] | [0.43-1.00] | [0.35-1.31] | [0.76-1.13] | [0.57-1.14] | [0.75-1.12] | [0.56-1.12] |
| Southeast | 0.97 | 0.84 | 1.89*** | 1.43 | 0.83 | 0.71* | 0.82 | 0.70* |
|  | [0.77-1.21] | [0.60-1.20] | [1.22-2.93] | [0.71-2.89] | [0.64-1.06] | [0.49-1.04] | [0.64-1.05] | [0.48-1.02] |
| Southwest | 0.91 | 0.86 | 1.28 | 1.41 | 0.82* | 0.70* | 0.82* | 0.70** |
|  | [0.75-1.11] | [0.62-1.19] | [0.87-1.88] | [0.74-2.69] | [0.66-1.02] | [0.49-1.00] | [0.66-1.02] | [0.49-0.99] |
| **Additional Eligibility Criteria** |  |  |  |  |  |  |  |  |
| Children and Families | 0.96 | 0.97 | 1.07 | 1.04 | 1.00 | 1.01 | 0.98 | 0.98 |
|  | [0.75-1.23] | [0.75-1.24] | [0.66-1.71] | [0.65-1.67] | [0.77-1.31] | [0.77-1.32] | [0.75-1.28] | [0.75-1.28] |
| Disabled | 0.92 | 0.92 | 1.14 | 1.14 | 0.89 | 0.89 | 0.88 | 0.87 |
|  | [0.72-1.19] | [0.71-1.18] | [0.71-1.84] | [0.71-1.84] | [0.68-1.17] | [0.67-1.17] | [0.67-1.16] | [0.66-1.15] |
| Healthy Horizons | 0.78 | 0.77 | 1.00 | 0.99 | 0.78 | 0.76 | 0.79 | 0.77 |
|  | [0.56-1.08] | [0.55-1.06] | [0.54-1.87] | [0.53-1.85] | [0.55-1.11] | [0.54-1.09] | [0.56-1.12] | [0.54-1.10] |
| Chronically Ill | 0.63 | 0.62 | - | - | 0.59 | 0.58 | 0.6 | 0.58 |
|  | [0.17-2.30] | [0.17-2.26] |  |  | [0.14-2.44] | [0.14-2.38] | [0.14-2.46] | [0.14-2.41] |
| **Total Enrollment Days Per Calendar Year** |  |  |  |  |  |  |  |  |
| 180 – <340 | REF | REF | REF | REF | REF | REF | REF | REF |
| >340 – <350 | 1.00 | 1.00 | 0.85 | 0.87 | 1.02 | 1.02 | 1.02 | 1.01 |
|  | [0.88-1.15] | [0.88-1.15] | [0.66-1.11] | [0.67-1.13] | [0.88-1.18] | [0.88-1.18] | [0.88-1.18] | [0.88-1.18] |
| >350 – <360 | 1.08 | 1.08 | 1.04 | 1.05 | 1.13* | 1.13* | 1.13* | 1.12* |
|  | [0.96-1.22] | [0.96-1.22] | [0.83-1.30] | [0.84-1.32] | [0.99-1.29] | [0.99-1.28] | [0.99-1.28] | [0.98-1.28] |
| >360 | 1.30*** | 1.30*** | 1.12 | 1.14 | 1.37*** | 1.37*** | 1.36*** | 1.36*** |
|  | [1.16-1.45] | [1.16-1.45] | [0.91-1.38] | [0.92-1.40] | [1.22-1.55] | [1.21-1.54] | [1.21-1.54] | [1.20-1.53] |
| **Total number of Chronic Conditions** |  |  |  |  |  |  |  |  |
| 0 | REF | REF | REF | REF | REF | REF | REF | REF |
| 1 – 2 | 1.67*** | 1.67*** | 1.37*** | 1.37*** | 1.73*** | 1.73*** | 1.73*** | 1.72*** |
|  | [1.51-1.85] | [1.50-1.85] | [1.13-1.67] | [1.13-1.67] | [1.55-1.94] | [1.54-1.93] | [1.54-1.93] | [1.54-1.93] |
| 3 – 4 | 2.31*** | 2.32*** | 1.98*** | 1.98*** | 2.40*** | 2.40*** | 2.40*** | 2.40*** |
|  | [2.04-2.62] | [2.04-2.63] | [1.56-2.50] | [1.56-2.51] | [2.09-2.75] | [2.09-2.75] | [2.09-2.75] | [2.09-2.75] |
| 5 or more | 2.81*** | 2.82*** | 2.14*** | 2.19*** | 2.87*** | 2.86*** | 2.85*** | 2.84*** |
|  | [2.44-3.25] | [2.44-3.26] | [1.62-2.82] | [1.66-2.89] | [2.45-3.35] | [2.45-3.34] | [2.43-3.33] | [2.43-3.32] |
| Missing | 0.32*** | 0.32*** | 0.48*** | 0.48*** | 0.30*** | 0.30*** | 0.30*** | 0.30*** |
|  | [0.28-0.37] | [0.28-0.37] | [0.36-0.64] | [0.37-0.64] | [0.25-0.35] | [0.25-0.35] | [0.25-0.35] | [0.25-0.35] |
| **Frequency of Primary Care Services Use per Calendar Year** |  |  |  |  |  |  |  |  |
| 0 | REF | REF | REF | REF | REF | REF | REF | REF |
| >0 – <2 | 9.17*** | 9.22*** | 17.41*** | 17.48*** | 8.09*** | 8.15*** | 8.17*** | 8.24*** |
|  | [7.73-10.87] | [7.77-10.93] | [11.01-27.51] | [11.05-27.64] | [6.68-9.79] | [6.73-9.87] | [6.74-9.90] | [6.80-9.99] |
| >2 – <4 | 3.33*** | 3.37*** | 6.99*** | 7.02*** | 3.02*** | 3.05*** | 3.04*** | 3.08*** |
|  | [2.76-4.03] | [2.78-4.07] | [4.28-11.39] | [4.30-11.46] | [2.44-3.73] | [2.47-3.78] | [2.46-3.76] | [2.49-3.81] |
| >4 | 1.68*** | 1.69*** | 3.38*** | 3.38*** | 1.59*** | 1.60*** | 1.60*** | 1.61*** |
|  | [1.38-2.05] | [1.39-2.06] | [2.03-5.61] | [2.03-5.61] | [1.27-1.97] | [1.29-1.99] | [1.28-1.99] | [1.29-2.01] |
| ***County-level Characteristics*** | | | | | | | | |
| **Median Household Income** |  | 1.00 |  | 1.00 |  | 1.00 |  | 1.00 |
|  |  | [1.00-1.00] |  | [1.00-1.00] |  | [1.00-1.00] |  | [1.00-1.00] |
| **Percent living under poverty** |  | 1.02 |  | 0.98 |  | 1.03 |  | 1.03 |
|  |  | [0.98-1.06] |  | [0.90-1.07] |  | [0.99-1.08] |  | [0.99-1.08] |
| **Percent unemployed, 16+** |  | 0.94 |  | 1.00 |  | 0.96 |  | 0.96 |
|  |  | [0.84-1.04] |  | [0.79-1.26] |  | [0.86-1.08] |  | [0.85-1.08] |
| **Percent <High School Diploma, 25+** |  | 1.01 |  | 0.94* |  | 1.03 |  | 1.03 |
|  |  | [0.98-1.05] |  | [0.88-1.01] |  | [0.99-1.06] |  | [0.99-1.06] |
| **Percent Non-Hispanic White** |  | 1.00 |  | 0.96*** |  | 1.01 |  | 1.01 |
|  |  | [0.99-1.01] |  | [0.94-0.99] |  | [1.00-1.02] |  | [0.99-1.02] |
| **Percent Living in Urban Areas** |  | 1.00 |  | 1.00 |  | 1.00 |  | 1.00 |
|  |  | [0.99-1.00] |  | [0.99-1.01] |  | [0.99-1.00] |  | [0.99-1.00] |
| **# of PCPs per 1000 persons** |  | 2.12** |  | 3.90** |  | 1.76 |  | 1.83* |
|  |  | [1.13-3.96] |  | [1.17-13.04] |  | [0.89-3.50] |  | [0.92-3.65] |
| **# of Specialists per 1000 persons** |  | 0.91 |  | 0.77** |  | 0.95 |  | 0.94 |
|  |  | [0.82-1.02] |  | [0.61-0.97] |  | [0.84-1.06] |  | [0.84-1.06] |
| **# of Short-term General Hospital Beds per 1000 persons** |  | 1.01 |  | 1.04 |  | 1.01 |  | 1.01 |
|  |  | [0.95-1.07] |  | [0.92-1.17] |  | [0.95-1.08] |  | [0.95-1.08] |
| **# of FQHCs per 1000 persons** |  | 0.44 |  | 0.40 |  | 0.59 |  | 0.61 |
|  |  | [0.07-2.77] |  | [0.01-17.48] |  | [0.08-4.30] |  | [0.08-4.43] |
| **# of RHCs per 1000 persons** |  | 1.30 |  | 0.03 |  | 2.00 |  | 2.09 |
|  |  | [0.14-12.01] |  | [0.00-5.14] |  | [0.19-21.48] |  | [0.19-22.54] |
| **CRC**=Colorectal Cancer; **MCO**=Managed Care Organizations; **PCP**=Primary Care Physicians; **FQHC**=Federally Qualified Health Centers; **RHC**=Rural Health Centers  **NOTE** Any CRC tests include any stool-based tests (gFOBT, FIT, or FIT-DNA) or any visual tests (standard colonoscopy, flexible sigmoidoscopy, or CT colonography). Enrollees who received any CRC tests include 393 enrollees who received both stool-based tests and visual tests during the study period.  Column (1) adjusts for individual-level characteristics only; Column (2) adjusts for both individual- and county-level charactersitics.  Regarding rurality of residence, we defined metro counties of <1 million population as counties in metro areas of 250,000 to 1 million population or counties in metro areas of fewer than 250,000 population. We defined non-metro counties as counties with urban population of 20,000 or more, adjacent to a metro area; urban population of 2,500 to 19,999, adjacent to a metro area; urban population of 2,500 to 19,990, not adjacent to a metro area; completely rural or less than 2,500 urban population, adjacent to a metro area; or, completely rural or less than 2,500 urban population, not adjacent to a metro area; and, urban areas with populations ranging from 2,500 to 49,999 that are not part of larger labor market areas.  Regarding frequency of primary care services, we translated multiple claims of primary care services on the same day into a single claim by prioritizing FQHCs/RHCs over other sites of care if they visited both FQHCs/RHCs and physician’s office, outpatient hospitals, independent clinics, or public health clinics; we prioritized non-physician’s office for the enrollees with multiple claims of primary care services on the same day if they visited both a physician’s office and outpatient hospitals, independent clinics, or public health clinics. We counted the number of claims for primary care services prior to the first claim for CRC testing for the enrollees who received any CRC testing as this study examines the association between frequency of primary care services use and CRC testing.  ***p<0.001, **p<0.01, *p<0.05 | | | | | | | | |

# Table S12. Weighted Associations (Odds Ratio [95% CI]) between Individual- and County-level Characteristics of Medicaid Enrollees and Receipt of CRC tests

|  | **Any CRC Tests** | | **Stool-based Tests Alone** | | **Visual Tests Alone** | | **Standard Colonoscopy** | |
| --- | --- | --- | --- | --- | --- | --- | --- | --- |
|  | **(1)** | **(2)** | **(1)** | **(2)** | **(1)** | **(2)** | **(1)** | **(2)** |
| ***Individual-level Characteristics*** | | | | | | | | |
| **Age** |  |  |  |  |  |  |  |  |
| 50-54 | REF | REF | REF | REF | REF | REF | REF | REF |
| 55-59 | 1.09 | 1.07 | 1.16 | 1.15 | 1.06 | 1.04 | 1.05 | 1.03 |
|  | [0.90-1.31] | [0.89-1.29] | [0.83-1.62] | [0.82-1.61] | [0.86-1.30] | [0.85-1.27] | [0.85-1.29] | [0.84-1.26] |
| 60-64 | 0.70** | 0.70** | 0.64** | 0.64** | 0.68*** | 0.67*** | 0.68*** | 0.68*** |
|  | [0.52-0.96] | [0.52-0.93] | [0.43-0.96] | [0.43-0.96] | [0.50-0.91] | [0.51-0.90] | [0.50-0.91] | [0.51-0.90] |
| **Male** | 1.47*** | 1.49*** | 1.51*** | 1.54*** | 1.41*** | 1.42*** | 1.41*** | 1.43*** |
|  | [1.23-1.76] | [1.25-1.77] | [1.14-2.00] | [1.16-2.05] | [1.16-1.70] | [1.18-1.72] | [1.17-1.71] | [1.18-1.72] |
| **Race/Ethnicity** |  |  |  |  |  |  |  |  |
| Non-Hispanic White | REF | REF | REF | REF | REF | REF | REF | REF |
| Non-Hispanic Black | 1.16 | 1.08 | 0.64* | 0.56** | 1.25* | 1.17 | 1.26* | 1.17 |
|  | [0.91-1.48] | [0.82-1.41] | [0.40-1.02] | [0.35-0.91] | [0.96-1.63] | [0.87-1.57] | [0.96-1.64] | [0.86-1.57] |
| Hispanic | 1.57** | 1.57** | 1.21 | 1.07 | 1.67*** | 1.69*** | 1.68*** | 1.70*** |
|  | [1.09-2.25] | [1.09-2.26] | [0.69-2.13] | [0.61-1.86] | [1.13-2.45] | [1.14-2.51] | [1.14-2.48] | [1.15-2.52] |
| Other | 1.63*** | 1.57*** | 1.66** | 1.54** | 1.59** | 1.53** | 1.61*** | 1.55** |
|  | [1.18-2.27] | [1.13-2.17] | [1.06-2.58] | [1.00-2.38] | [1.11-2.26] | [1.07-2.20] | [1.13-2.30] | [1.08-2.23] |
| **Rurality of residence** |  |  |  |  |  |  |  |  |
| Metro counties  >1 million population | REF | REF | REF | REF | REF | REF | REF | REF |
| Metro counties  <1 million population | 0.91 | 0.99 | 0.94 | 1.14 | 0.92 | 0.89 | 0.92 | 0.89 |
|  | [0.61-1.35] | [0.58-1.68] | [0.55-1.60] | [0.55-2.36] | [0.60-1.41] | [0.50-1.57] | [0.60-1.42] | [0.51-1.58] |
| Nonmetro counties | 0.84 | 0.77 | 1.05 | 2.95** | 0.85 | 0.67 | 0.86 | 0.67 |
|  | [0.58-1.22] | [0.43-1.36] | [0.59-1.89] | [1.29-6.73] | [0.57-1.29] | [0.36-1.25] | [0.57-1.30] | [0.36-1.26] |
| **MCO regions** |  |  |  |  |  |  |  |  |
| Lehigh Capital | REF | REF | REF | REF | REF | REF | REF | REF |
| New East | [0.71-1.43] | [0.77-2.11] | [0.66-1.51] | [0.52-2.02] | [0.74-1.53] | [0.69-2.23] | [0.74-1.53] | [0.69-2.25] |
|  | 1.15 | 1.18 | 1.16 | 1.05 | 1.24 | 1.16 | 1.24 | 1.17 |
| New West | [0.77-1.71] | [0.59-2.38] | [0.52-2.60] | [0.39-2.79] | [0.82-1.87] | [0.52-2.60] | [0.82-1.87] | [0.52-2.63] |
|  | 0.73 | 1.14 | 2.33** | 1.39 | 0.64* | 0.85 | 0.63* | 0.80 |
| Southeast | [0.46-1.17] | [0.53-2.47] | [1.22-4.45] | [0.48-4.00] | [0.38-1.05] | [0.39-1.84] | [0.38-1.04] | [0.37-1.74] |
|  | 0.82 | 0.90 | 1.56* | 1.66 | 0.78 | 0.73 | 0.77 | 0.74 |
| Southwest | [0.55-1.22] | [0.46-1.77] | [0.94-2.59] | [0.62-4.43] | [0.51-1.18] | [0.33-1.59] | [0.51-1.17] | [0.34-1.62] |
|  | [0.71-1.43] | [0.77-2.11] | [0.66-1.51] | [0.52-2.02] | [0.74-1.53] | [0.69-2.23] | [0.74-1.53] | [0.69-2.25] |
| **Additional eligibility criteria** |  |  |  |  |  |  |  |  |
| Children and Families | 1.03 | 1.04 | 0.67 | 0.67 | 1.16 | 1.16 | 1.13 | 1.13 |
|  | [0.62-1.70] | [0.64-1.68] | [0.39-1.15] | [0.39-1.15] | [0.66-2.02] | [0.68-1.99] | [0.64-2.00] | [0.66-1.96] |
| Disabled | 1.05 | 1.07 | 1.28 | 1.19 | 1.06 | 1.08 | 1.06 | 1.09 |
|  | [0.66-1.69] | [0.68-1.68] | [0.62-2.63] | [0.58-2.42] | [0.64-1.76] | [0.67-1.76] | [0.64-1.77] | [0.67-1.79] |
| Healthy Horizons | 0.84 | 0.82 | 1.27 | 1.23 | 0.82 | 0.79 | 0.83 | 0.8 |
|  | [0.51-1.38] | [0.51-1.33] | [0.56-2.88] | [0.53-2.84] | [0.47-1.43] | [0.46-1.36] | [0.48-1.46] | [0.47-1.37] |
| Chronically Ill | 0.23* | 0.21* | - | - | 0.29 | 0.26 | 0.29 | 0.26 |
|  | [0.04-1.27] | [0.04-1.17] |  |  | [0.05-1.73] | [0.04-1.55] | [0.05-1.74] | [0.04-1.55] |
| **Total enrollment days per calendar year** |  |  |  |  |  |  |  |  |
| 180 – <340 | REF | REF | REF | REF | REF | REF | REF | REF |
| >340 – <350 | 0.79 | 0.80 | 0.68 | 0.69 | 0.81 | 0.82 | 0.80 | 0.81 |
|  | [0.58-1.08] | [0.59-1.09] | [0.40-1.17] | [0.40-1.19] | [0.57-1.15] | [0.58-1.15] | [0.57-1.13] | [0.57-1.14] |
| >350 – <360 | 0.9 | 0.9 | 0.83 | 0.84 | 0.96 | 0.95 | 0.96 | 0.95 |
|  | [0.68-1.20] | [0.68-1.18] | [0.52-1.31] | [0.53-1.34] | [0.70-1.32] | [0.70-1.29] | [0.70-1.31] | [0.70-1.29] |
| >360 | 1.26 | 1.26 | 1.03 | 1.05 | 1.28 | 1.28 | 1.26 | 1.26 |
|  | [0.97-1.63] | [0.98-1.63] | [0.68-1.58] | [0.68-1.61] | [0.96-1.70] | [0.97-1.69] | [0.95-1.67] | [0.95-1.66] |
| **Total number of chronic conditions** |  |  |  |  |  |  |  |  |
| 0 | REF | REF | REF | REF | REF | REF | REF | REF |
| 1 – 2 | 2.15*** | 2.13*** | 1.66** | 1.66** | 2.37*** | 2.35*** | 2.32*** | 2.30*** |
|  | [1.68-2.75] | [1.67-2.72] | [1.07-2.56] | [1.08-2.56] | [1.82-3.08] | [1.81-3.05] | [1.79-3.02] | [1.77-2.99] |
| 3 – 4 | 3.19*** | 3.21*** | 2.50*** | 2.50*** | 3.60*** | 3.64*** | 3.58*** | 3.62*** |
|  | [2.31-4.40] | [2.34-4.41] | [1.54-4.07] | [1.52-4.10] | [2.57-5.04] | [2.61-5.07] | [2.55-5.02] | [2.59-5.05] |
| 5 or more | 3.54*** | 3.52*** | 1.97*** | 2.02*** | 3.98*** | 3.94*** | 3.90*** | 3.85*** |
|  | [2.53-4.95] | [2.54-4.88] | [1.29-3.01] | [1.33-3.09] | [2.78-5.72] | [2.77-5.61] | [2.72-5.61] | [2.70-5.49] |
| Missing | 0.34*** | 0.34*** | 0.54*** | 0.55*** | 0.34*** | 0.33*** | 0.34*** | 0.33*** |
|  | [0.28-0.42] | [0.28-0.41] | [0.37-0.79] | [0.38-0.79] | [0.27-0.42] | [0.27-0.41] | [0.27-0.42] | [0.26-0.41] |
| **Frequency of primary care services use per calendar year** |  |  |  |  |  |  |  |  |
| 0 | REF | REF | REF | REF | REF | REF | REF | REF |
| >0 – <2 | 3.92*** | 3.96*** | 6.57*** | 6.57*** | 3.61*** | 3.63*** | 3.65*** | 3.68*** |
|  | [3.16-4.87] | [3.21-4.89] | [3.89-11.10] | [3.88-11.11] | [2.86-4.55] | [2.88-4.57] | [2.89-4.61] | [2.92-4.64] |
| >2 – <4 | 1.36** | 1.38** | 2.71*** | 2.78*** | 1.26 | 1.27 | 1.28* | 1.29* |
|  | [1.04-1.77] | [1.06-1.79] | [1.49-4.91] | [1.54-5.03] | [0.95-1.69] | [0.95-1.70] | [0.96-1.71] | [0.97-1.73] |
| >4 | 0.57*** | 0.57*** | 1.25 | 1.26 | 0.53*** | 0.53*** | 0.54*** | 0.54*** |
|  | [0.42-0.77] | [0.43-0.77] | [0.66-2.36] | [0.66-2.38] | [0.38-0.74] | [0.38-0.74] | [0.39-0.75] | [0.39-0.75] |
| ***County-level Characteristics*** | | | | | | | | |
| **Median household income** |  | 1.00* |  | 1.00 |  | 1.00 |  | 1.00 |
|  |  | [1.00-1.00] |  | [1.00-1.00] |  | [1.00-1.00] |  | [1.00-1.00] |
| **Percent living in urban areas** |  | 1.00 |  | 1.00 |  | 1.00 |  | 1.00 |
|  |  | [0.99-1.01] |  | [0.99-1.02] |  | [0.99-1.01] |  | [0.99-1.01] |
| **Percent Non-Hispanic White** |  | 1.00 |  | 0.95*** |  | 1.02 |  | 1.02 |
|  |  | [0.98-1.03] |  | [0.92-0.98] |  | [0.99-1.04] |  | [0.99-1.04] |
| **Percent living under poverty** |  | 0.99 |  | 1.00 |  | 1.01 |  | 1.02 |
|  |  | [0.90-1.08] |  | [0.88-1.14] |  | [0.91-1.12] |  | [0.92-1.13] |
| **Percent unemployed among population of 16+ years** |  | 0.74** |  | 0.88 |  | 0.78* |  | 0.78* |
|  |  | [0.58-0.95] |  | [0.58-1.34] |  | [0.60-1.02] |  | [0.60-1.02] |
| **Percent <high school diploma among population of 25+ years** |  | 1.00 |  | 0.89** |  | 1.02 |  | 1.03 |
|  |  | [0.93-1.07] |  | [0.79-1.00] |  | [0.95-1.10] |  | [0.95-1.11] |
| **# of PCPs per 1000 persons** |  | 0.68 |  | 1.94 |  | 0.73 |  | 0.72 |
|  |  | [0.16-2.95] |  | [0.37-10.11] |  | [0.16-3.34] |  | [0.16-3.30] |
| **# of specialists per 1000 persons** |  | 1.19 |  | 0.73* |  | 1.22 |  | 1.23 |
|  |  | [0.93-1.52] |  | [0.51-1.05] |  | [0.95-1.55] |  | [0.96-1.56] |
| **# of short-term general hospital beds per 1000 persons** |  | 0.92 |  | 1.13 |  | 0.91 |  | 0.91 |
|  |  | [0.82-1.04] |  | [0.96-1.33] |  | [0.80-1.03] |  | [0.80-1.03] |
| **# of FQHCs per 1000 persons** |  | 0.92 |  | 21.31 |  | 1.97 |  | 2.19 |
|  |  | [0.01-80.44] |  | [0.01-56,239.60] |  | [0.02-202.29] |  | [0.02-224.52] |
| **# of RHCs per 1000 persons** |  | 7.45 |  | 0.02 |  | 21.66 |  | 23.59 |
|  |  | [0.09-645.75] |  | [0.00-22.67] |  | [0.18-2,625.66] |  | [0.19-2,886.43] |
| **CRC**=Colorectal Cancer; **MCO**=Managed Care Organizations; **PCP**=Primary Care Physicians; **FQHC**=Federally Qualified Health Centers; **RHC**=Rural Health Centers  **NOTE** All estimates were weighted by a composite inverse probability weighting score based on an enrollee’s propensity to enroll longer in Medicaid and to use primary care services during the study period and adjusted for all individual- and county-level characteristics.  Any CRC tests include any stool-based tests (gFOBT, FIT, or FIT-DNA) or any visual tests (standard colonoscopy, flexible sigmoidoscopy, or CT colonography). Enrollees who received any CRC tests include 393 enrollees who received both stool-based tests and visual tests during the study period.  Column (1) adjusts for individual-level characteristics only; Column (2) adjusts for both individual- and county-level charactersitics.  Regarding rurality of residence, we defined metro counties of <1 million population as counties in metro areas of 250,000 to 1 million population or counties in metro areas of fewer than 250,000 population. We defined non-metro counties as counties with urban population of 20,000 or more, adjacent to a metro area; urban population of 2,500 to 19,999, adjacent to a metro area; urban population of 2,500 to 19,990, not adjacent to a metro area; completely rural or less than 2,500 urban population, adjacent to a metro area; or, completely rural or less than 2,500 urban population, not adjacent to a metro area; and, urban areas with populations ranging from 2,500 to 49,999 that are not part of larger labor market areas.  Regarding frequency of primary care services, we translated multiple claims of primary care services on the same day into a single claim by prioritizing FQHCs/RHCs over other sites of care if they visited both FQHCs/RHCs and physician’s office, outpatient hospitals, independent clinics, or public health clinics; we prioritized non-physician’s office for the enrollees with multiple claims of primary care services on the same day if they visited both a physician’s office and outpatient hospitals, independent clinics, or public health clinics. We counted the number of claims for primary care services prior to the first claim for CRC testing for the enrollees who received any CRC testing as this study examines the association between frequency of primary care services use and CRC testing.  ***p<0.001, **p<0.01, *p<0.05 | | | | | | | | |

1.
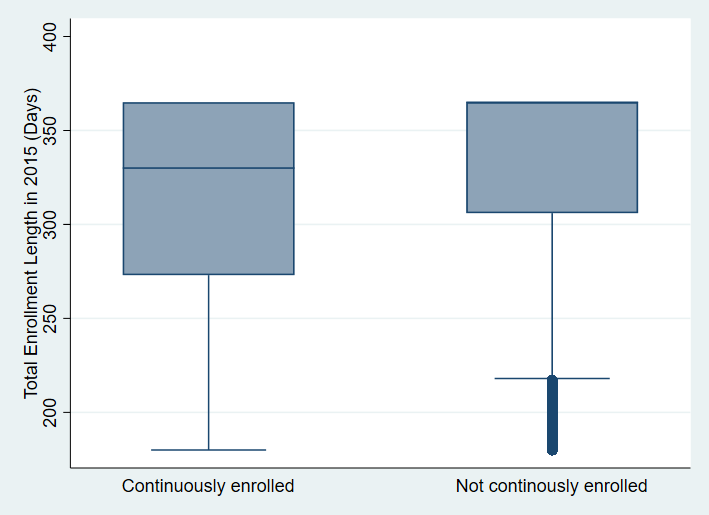
 [↑](#footnote-ref-1)
2. (n_c_-n_d_)/t where n_c_ is the number of pairs that are concordant, n_d_ the number of pairs that are discordant, and t is the number of total number of pairs with different responses. [↑](#footnote-ref-2)
3. If a pair of observations with different responses is neither concordant nor discordant, it is a tie. [↑](#footnote-ref-3)
4. the ratio of the difference between the number of concordant pairs and the number of discordant pairs to the number of possible pairs (2(n_c_-n_d_)/(N(N-1)). [↑](#footnote-ref-4)
5. (n_c_-n_d_)/t where n_c_ is the number of pairs that are concordant, n_d_ the number of pairs that are discordant, and t is the number of total number of pairs with different responses. [↑](#footnote-ref-5)
6. If a pair of observations with different responses is neither concordant nor discordant, it is a tie. [↑](#footnote-ref-6)
7. the ratio of the difference between the number of concordant pairs and the number of discordant pairs to the number of possible pairs (2(n_c_-n_d_)/(N(N-1)). [↑](#footnote-ref-7)
